# The field factor: Industry publishing contribution and novelty in science

**DOI:** 10.1371/journal.pone.0346227

**Published:** 2026-04-27

**Authors:** Anubha Shokhand, Nilam Kaushik, Satyam Mukherjee

**Affiliations:** 1 Strategy, Indian Institute of Management Bangalore, Bengaluru, India; 2 School of Management and Entrepreneurship, Shiv Nadar Institution of Eminence, Greater Noida, India; Universita degli Studi di Foggia, ITALY

## Abstract

Firms frequently publish scientific articles as part of their R&D initiatives, motivated by commercial objectives. However, the extent of industry involvement in publishing varies across different scientific fields and can have implications for research within those fields. Novelty in science is associated with scientific and technological breakthroughs. In this paper, we examine a field-level antecedent of novelty—the extent of industry publishing contribution to a field—and its association with two key aspects of recombinant novelty of publications: the occurrence of a novel recombination (*novelty occurrence*), and the degree of novelty, captured through *novelty breadth*, reflecting the scope of novel integration of knowledge elements, and *novelty distance*, reflecting the extremity of conceptual divergence among novel knowledge recombinations. Drawing on a longitudinal dataset of 11.1 million publications across 1639 STEM fields from 2000 to 2014, we find that greater industry publishing contribution within a scientific field is associated with higher odds of *novelty occurrence* and greater *novelty breadth*, but lower *novelty distance*. Notably, university is an important driver of our results across the three dimensions of novelty highlighting the importance of industry publishing contribution in shaping the novelty of entire fields. In addition, we find that top-ranked research institutions appear better able to manage the trade-off between *novelty distance* and other forms of novelty as industry publishing contribution increases. Our findings emphasize the need for policymakers in encouraging and preserving more exploratory forms of novelty in fields with substantial industry publishing, where such exploration is particularly valuable.

## Introduction

Originality and novelty are important for advancing future science and technology through knowledge breakthroughs [1–3]. Novelty in scientific research also underpins recognition and rewards within the scientific community [4]. However, publishing novel scientific research is risky [2] and difficult [2,5,6]. This underscores the importance of exploring the drivers of recombinant novelty in science.

Scientific research has traditionally been anchored in universities [4], yet industrial firms have long complemented academic science by regularly publishing their research findings. For instance, industry-authored publications account for a quarter of the scientific publications in Artificial Intelligence [7] and in fields related to Biomedical research [8]. This pattern is notable despite the reported decline in “corporate science” by large corporations [8] across a range of industries in the United States due to a reduction in private benefits to research.

Unlike universities, however, firms publish under commercially driven objectives [9,10], and publications represent a strategic choice shaped by trade-offs between the benefits of openness and the need to appropriate knowledge commercially [9,11,12]. Industry participation in publishing reflects these underlying trade-offs, which may vary systematically across scientific fields, leading to heterogeneity in the extent of industry contribution to scientific publications across fields.

Because of industry’s commercial objectives, a greater extent of publishing within a field can reshape novelty by steering research towards use-inspired problems and commercially driven agendas [13–16], encouraging secrecy in academia [17–21]. Scientific fields characterized by higher levels of industry publishing, often reflecting stronger industry–university interactions, may exhibit systematically different patterns of novelty than those with lower industry publishing, motivating a field-level examination of industry publishing as a driver of publication novelty. The question of how industry involvement in publishing shapes the novelty of publications across scientific fields, is particularly germane given the growing convergence of university and industry in the production of scientific research, alongside industry’s commercially-driven research [22,23].

A dominant perspective rooted in the tradition of recombinant innovation conceptualizes novelty as unusual recombinations of antecedent knowledge [24]. Building on this view, scientific journals can be viewed as bodies of knowledge, and novelty is calculated by analyzing the distribution and co-occurrence patterns of journals cited in the bibliography of a scientific publication. Using a dataset of 11.1 million publications across 1639 STEM fields from 2000 to 2014, this paper explores the relationship between the extent of industry contribution to scientific publishing within a field and the novelty of publications within that field. After establishing the main relationship between industry publishing contribution and novelty, we conduct additional analyses to understand the underlying mechanisms. Firstly, to assess if the relationship persists across the entire field, we examine whether the association between industry publishing contribution [24–28] and novelty is driven primarily by university- or industry-authored publications within each field. Additionally, since highly ranked research institutions often possess stronger research infrastructure and more stable funding, they may respond differently to industry publishing within a field compared to others. Therefore, secondly, we explore whether the relationship between industry publishing contribution and novelty differs for publications affiliated with high-ranking compared to other research institutions.

This work sheds light on the role of industry engagement in shaping scientific research within entire fields by unpacking how the extent of industry publishing contribution relates to different dimensions of novelty across fields. Our findings also have important implications for science policy, providing guidance for policymakers in designing more targeted and effective university-industry (UI) engagement policies.

## Theory

### Industry contribution to scientific publishing

Industry firms regularly conduct and publish scientific research for multiple strategic reasons [9], including accessing external knowledge and resources, attracting and retaining scientific talent [10,11,29], building absorptive capacity [11,30–32], supporting downstream innovation [12,31,33], enhancing firm reputation, and gaining legitimacy and trust from stakeholders [10,31]. At the same time, scientific publications are a public good that is broadly accessible beyond firm boundaries [34], potentially reducing appropriability and creating a tension between the benefits of openness and the need to capture commercial value from knowledge [9,18,31].

Since this openness-appropriation trade-off may vary across scientific fields, industry’s share of publishing can differ substantially from field to field. Industry publishing is particularly prominent in Pasteur’s Quadrant fields, such as Biotechnology, Computer Science, and Artificial Intelligence, where advances in scientific research are tightly coupled with technological innovation [35]. Therefore, industry firms’ engagement with the scientific frontier in Pasteur’s Quadrant fields can directly support the firms’ downstream innovation [31]. Although firms may invest in basic research to build absorptive capacity [31], firms largely tend to publish more in applied fields than in basic fields, where scientific output often has more apparent near-term relevance for problem solving and technological innovation [17,18,36,37]. Since applied research is closer to commercialization, firms can more readily capture private returns through commercialization and complementary downstream capabilities, making openness less expensive. In contrast, in basic fields, where advances are farther from application, scientific research in those fields may generate weaker immediate private returns relative to the cost of openness. Firms may also publish in fields associated with controversial products or technologies (e.g., genetically modified crops in Biotechnology, deepfakes, and facial recognition in Artificial Intelligence) to demonstrate responsible conduct and strengthen trust among external stakeholders [13]. In such fields, commercialization is not hindered because firms can maintain a commercial advantage through IP protection and downstream capabilities, and publishing disclosures may relate to foundational, methodological, or normative components rather than the most commercially sensitive components of their innovations [13]. Additionally, firms are especially likely to publish in fields that generate theoretical foundations for how technological components function and interact, helping firms make sense of uncertain technologies and guide subsequent problem solving and recombination [23,38]. For instance, research by firms on the structure of cell receptors helped scientists design molecules that could bind to the receptor sites and activate or inhibit the functioning of specific body cells, and led to the development of the drug Prozac, which selectively inhibits the action of specific brain cell receptors, which helps treat anxiety and depression [39].

### Measuring novelty

Despite the practical relevance of understanding the drivers of novel science, no single novelty measurement approach or indicator captures all its dimensions [40], and relatively few studies have systematically examined the multifaceted nature of recombinant novelty [40–43]. Existing approaches of measuring novelty can broadly be grouped into four categories, each reflecting distinct aspects of the nature and magnitude of novelty. Categorical measures operationalize novelty in binary terms, indicating whether a previously unseen knowledge component or combination is present or absent [44,45], configurational approaches capture the degree of distance or divergence in features [46,47], structural measures adopt a network perspective, emphasizing the position of recombined knowledge in the broader knowledge network [45,48], and combinatorial measures operationalize novelty as arising from rare combinations [2,3,45,49,50].

We follow the combinatorial perspective of novelty, which views novelty as unconventional recombinations of prior knowledge elements [2,3,49]. In the context of scientific publications, novelty has primarily been operationalized by examining the distribution and co-occurrence of patterns of referenced journals in the bibliography of publications [2,3]. Some publications incorporate mildly atypical combinations of journals that, while novel, remain adjacent to dominant knowledge streams. In contrast, others integrate highly disparate or rarely connected domains, indicating a deeper level of conceptual innovation [2,3,51]. Novel recombinations can also vary in scope, ranging from broad integrations that span multiple ideas or disciplines to narrow conceptual shifts [2]. These differences in the conceptual distance and scope of recombined knowledge streams align with Guilford’s conceptualisation [52] of the creative production processes of *originality* (the statistical rarity of responses) and *fluency* (total responses generated). These patterns of conceptual distances and the scope of recombined knowledge streams provide a basis for quantifying the degree of novelty in scientific work.

However, atypical recombinations require cognitive leaps and significant departures from established knowledge [2,3,49]. As a result, most publications make incremental advances, and only a small proportion exhibit novel recombinations [2,3]. Therefore, the occurrence of novel recombinations is rare and may confound the patterns for the degree of novelty. This rarity of novel recombinations is especially salient in our field-level analysis, where aggregate patterns may mask whether the observed effects are driven by the frequency of novelty occurrences or by the degree of novelty of publications within fields. Therefore, in this work, we examine the occurrence of novelty in addition to the degree of novelty.

***Novelty occurrence***: *Novelty occurrence* of a publication captures whether a publication involves an unexpected recombination of knowledge and indicates a new cognitive leap, and is consistent with the categorical operationalization of novelty [44,45].

**Degree of Novelty**: Consistent with Guilford’s (1976) [52] view, we characterize the degree of novelty along two dimensions: *novelty breadth*, which captures the scope of knowledge recombinations, and *novelty distance*, which captures the extremities of cognitive distance spanned by those recombinations.

***Novelty breadth*** refers to the overall scope of novel recombinations within a publication. It reflects the extent to which diverse and previously unconnected knowledge components are brought together. We operationalize this by summing the intellectual distances of all novel knowledge combinations, thereby capturing the cumulative novelty embedded in the recombination process. Consequently, higher *novelty breadth* reflects a publication’s engagement with a wide range of conceptually diverse domains through novel recombinations.

***Novelty distance***, in contrast, captures the extremity of conceptual divergence among the recombined knowledge elements, following Koestler’s conceptualisation of creative bisociation as the intersection of separate thought matrices [53]. Specifically, it measures the intellectual distance between the two most conceptually distant knowledge components within a publication’s set of novel recombinations. This dimension reflects the difficulty of integrating the most disparate ideas, highlighting a publication’s capacity for radical knowledge integration.

Both aspects of novelty, *novelty occurrence* and the degree of novelty (*novelty breadth* and *novelty distance*), are distinct dimensions and are linked to knowledge breakthroughs. *Novelty occurrence* indicates a new cognitive leap and is linked to breakthrough publications [2]. Many transformative scientific advances have emerged from a broad scope of novel recombinations, i.e., high *novelty breadth* [2]. A representative example is the study of the self-organization of Microtubules, which integrates concepts from Physics, Chemistry, and Biology to explain cellular formation processes [54]. This kind of interdisciplinary recombination demonstrates how a broad scope of recombinations can advance scientific understanding by connecting several disparate ideas. Several groundbreaking scientific breakthroughs have also emerged by recombining conceptually distant ideas—i.e., high *novelty distance*—often originating from curiosity-driven inquiry. For instance, Chaos Theory emerged from Edward Lorenz’s curiosity-driven exploration of nonlinear equations in the context of weather prediction, leading to profound implications across disciplines [55]. Similarly, Prospect Theory was developed through curiosity-driven research by Daniel Kahneman and Amos Tversky by recombining ideas from Economics and Psychology. Such curiosity-driven research or *science for the sake of science* is an important aspect of the scientific enterprise [4].

Since these dimensions of novelty embody distinct characteristics, their associations with antecedents and their impacts may be complex and multifaceted. We explore how industry publishing contribution to a field relates to (*novelty occurrence*) and the degree of novelty captured by *novelty breadth* and *novelty distance* of publications within the field (Table 1).

**Table 1:** Dimensions of Novelty examined in this study.

Novelty Dimension	Description	Operationalization
***Novelty occurrence***	Indicates whether a publication involves an unexpected recombination of prior knowledge, reflecting the presence of a cognitive leap.	Binary indicator equal to 1 if a publication contains at least one novel journal combination.
***Degree of Novelty***		
*Novelty breadth*	Scope of novel combinations, reflecting how widely knowledge is recombined by capturing the cumulative novelty across the novel combinations.	Sum of intellectual distances across all the novel journal combinations within the publication.
*Novelty distance*	Extremity of conceptual divergence across the novel combinations.	Intellectual distance between the two most conceptually distant knowledge components in the set of all novel journal combinations within the publication.

### Industry contribution to a field and novelty

Scientific research is organized into “fields,” which are research communities with shared interests, goals, norms of language, approaches, and institutional environments to knowledge creation [56,57]. These community-level differences shape different aspects of knowledge created within these fields, such as novelty, impact, and collaboration patterns [58].

Since university research is primarily driven by scientific curiosity and the academic reward system, while industry research is primarily shaped by commercial objectives, scientific research produced by these two entities often differs [18,23,36,59–61]. For instance, university typically pursue long-term basic research, whereas industry tends to prioritize short-term, application-oriented research aligned with market needs [18,36,61]. A high degree of industry contribution to publishing within a field often signals greater interaction and knowledge exchange between industry and university. Since there are differences in research by university and industry, a greater extent of industry contribution to a field may have important implications for the novelty of scientific publications within the whole field.

Industry tends to focus on practical and complex scientific problems [36,62], frequently requiring interdisciplinary research and diverse expertise [63]. Such a setting could enable novel recombinations as scientists from different backgrounds collaborate [63], potentially forging unexpected links between theories or paradigms [23]. This process can introduce a broader and more distant range of ideas into a field. Industry publications may sometimes themselves exhibit novel knowledge combinations [22,64–66], thereby introducing new ideas and approaches into a field. For example, Karl Jansky’s breakthrough discovery of extraterrestrial radio emissions, while investigating radio interference at Bell Laboratories, later stimulated extensive research in Radioastronomy [31]. Even when they do not display high novelty, the problems they introduce are often motivated by practical constraints and commercial applications, which may stimulate novel recombinations in follow-on research in the field. For example, semiconductor firms documented several engineering challenges associated with transistor scaling and the limits of Moore’s Law in scientific publications [67]. These discussions highlighted practical constraints in device performance and fabrication, which subsequently stimulated academic research on alternative transistor architectures, materials, and scaling approaches, many of which were developed by university researchers [67]. As a result, greater industry presence in a field can encourage novel recombinations by expanding the idea pool and offering new problems and perspectives that scientists can integrate with existing knowledge, thereby increasing the propensity for the occurrence of novel recombinations in their publications.

***H1:*** Industry publishing contribution to a field has a positive relationship with the *novelty occurrence* of publications within the field.

While industry publishing contribution to a field can provide a larger pool of ideas driving the propensity for novel recombinations, it could also drive the magnitude and nature of novelty through other mechanisms. To explore this, we focus on two key dimensions of *novelty breadth* and *novelty distance* that capture distinct yet complementary aspects of how knowledge elements are recombined in scientific research:

Greater industry contribution to a field is associated with greater industry influence on the field that can shape the direction of scientific inquiry by steering research toward commercially-driven agendas [13–17,68]. On the one hand, research driven by commercial agendas can stimulate work on complex, real-world problems that often require interdisciplinary collaboration and the integration of diverse knowledge domains [36,62,63]. This may encourage researchers within the field to pursue broad-scope novel recombinations spanning several knowledge elements, thereby increasing *novelty breadth* in fields with high industry publishing activity.

On the other hand, growing industry influence can narrow research to topics of interest to industry firms, limiting the diversity of research within a field. For instance, in the field of Artificial Intelligence, studies show a narrowing of research focus over time, with increased alignment to commercially lucrative areas [69,70]. Extensive industry’s incentives to patent commercially valuable discoveries may also promote secrecy [17,71–74], delay the publication of novel findings [18], and shift the locus of disclosure from academic publications to patents [17–21]. Such dynamics contribute to the “marketization” of science [75], potentially restricting the free flow of knowledge and slowing collective progress. This could lead to a decrease in *novelty breadth* of publications within the field. Therefore, the net effect of industry publishing contribution on *novelty breadth* of publications within a field is ambiguous and warrants empirical examination.

***H2a:*** Industry publishing contribution to a field has a positive relationship with the *novelty breadth* of publications within the field.
***H2b:*** Industry publishing contribution to a field has a negative relationship with the *novelty breadth* of publications within the field.

Several scientific breakthroughs have emerged from curiosity-driven blue-sky research that recombines highly intellectually distant knowledge. Such research is characterized by high *novelty distance*, is inherently risky [58], and may have commercial relevance only in the long term. Although greater industry involvement in fields may promote broad, novel recombinations that can help solve complex applied problems that require interdisciplinary approaches, it may simultaneously divert academic attention in those fields from exploratory research driven by pure scientific curiosity. As a result, although the breadth of novel recombinations may increase with the rise in industry publishing intensity within a field, it could simultaneously discourage the depth of intellectual distance of novel recombinations (*novelty distance*). Therefore, the benefit of increased scope for novel recombinations in fields with greater industry publishing contribution may be accompanied by diminished *novelty distance* of publications within the field.

Additionally, similar to *novelty breadth*, greater industry influence within a field could lead to a decrease in *novelty distance* of publications by steering research in specific directions and encouraging secrecy within the field.

***H3:*** Industry publishing contribution to a field has a negative relationship with the *novelty distance* of publications within the field.

In addition to testing these hypotheses, we examine whether the observed effects are driven by university or industry-authored publications and whether they vary for top-ranked research institutions. This extension allows us to assess heterogeneity in the mechanisms underlying our hypotheses and to draw implications for science policy.

## Empirical setting

Our dataset includes a pooled cross-section of 11.1 million scientific publications from journals in STEM fields published between 2000 and 2014 and indexed in OpenAlex [76]. OpenAlex data is publicly available, and we use the April 2024 bulk download version (https://docs.openalex.org/download-all-data/download-to-your-machine). OpenAlex is one of the most comprehensive databases of scientific publications [77], having equivalent coverage of information as its predecessor MAG [78] and with other popular sources such as Web of Science and Scopus [77,79]. We adopt this time window to ensure comparability with the novelty measure proposed by Wang et al. (2017) [2], which is calculated using publication data from the early 2000s (specifically 2001). Beginning the sample period around this time and extending it over a 15-year horizon allows for consistency with their approach while capturing longer-run patterns.

We use OpenAlex-assigned “concepts”, which are abstract ideas associated with a publication, to classify each publication into its corresponding scientific field (https://docs.openalex.org/api-entities/concepts). A hierarchy of concepts, Levels 0–3, is assigned to each publication in the dataset. 19 Level 0 concept codes represent broader research areas in publications, such as Physics and Chemistry. We filter publications in STEM fields if the Level 0 concept belongs to one of ‘Biology’, ‘Chemistry’, ‘Computer science’, ‘Engineering’, ‘Environmental science’, ‘Geology’, ‘Materials science’, ‘Mathematics’, ‘Medicine’, ‘Physics’, or ‘Psychology’. There are 284 Level 1 codes embedded within Level 0 concepts that represent narrower research fields for publications, such as ‘Structural Engineering’, ‘Nuclear Physics’, and ‘Machine Learning’. We use a combination of Level 0 and Level 1 concept codes to represent a field [80,81] (see SI Appendix S1 for details). Since a field refers to a scientific community interacting with others, highly granular Levels 3 and 4 OpenAlex concepts are too specific for our field-level analysis. A combination of 11 Level 0 concepts and 284 Level 1 concepts yields 1,639 fields and 19,251 field-years in our data. We have field size in the denominator for the measure of industry publishing contribution, a key independent variable in this work. A small field size could bias the measure of industry publishing contribution upwards. Therefore, we drop fields with fewer than 25 publications..

There are 17,516,004 journal publications in 1,639 STEM fields, of which we filtered 11,147,008 publications that we could classify into ‘university’ or ‘industry’ based on the institution-type keywords provided by OpenAlex for each author, and for which the journal impact factor can be calculated. We include Heckman’s missing variable correction [82] in our estimates to account for potential bias due to missing author affiliation. We have described the methodology for classifying each author’s affiliation type as ‘university’ or ‘industry’ in detail in SI Appendix S2.

As our research question is at the field level and since scientific fields evolve, we measure all the field variables at the field-year level.

### Dependent variable: Novelty

Consistent with a recombinant perspective, we conceptualize novelty as arising from unconventional combinations of prior knowledge elements. Since our research question is situated at the field level, we require a measure of the knowledge elements available for recombination that is comparable across fields. The set of journals cited in a publication provides such a metric. Therefore, we use journal combinations as a proxy for knowledge recombinations, employing the measures of recombinant novelty as outlined by Wang et al. (2017) [2].

We examine two facets of a publication’s novelty: *novelty occurrence*, and the degree of novelty captured through *novelty breadth*, indicating the scope of novel recombinations, and *novelty distance*, indicating the extremity of conceptual divergence among recombined knowledge.

***Novelty occurrence*** (𝒩ovelty𝒪ccurrence )

A novel combination of journals in a publication signifies a novel recombination of existing knowledge (*novelty occurrence*). We identify ‘novel pairs’ of journals by filtering journal pairs in the publication’s references that appear for the first time in the 20 years leading up to the publication year. To incorporate only ‘useful’ novel recombinations, we include journal pairs that have been cited in at least one publication in the following three years. Furthermore, to ensure that each journal combination represents a substantive and credible instance of knowledge recombination, we include only journal combinations in which both journals fall above the 75^*th*^ percentile of the journal impact factor distribution for that year. This threshold helps in focusing on recombinations involving widely recognized sources of scientific knowledge. The 75^*th*^ percentile journal impact factor cutoff varies between 0.80 and 1.35 over the 15-year period.

𝒩ovelty𝒪ccurrence  takes a value of one if a publication has a novel journal pair and zero otherwise.

### Degree of novelty

To evaluate the degree of novelty, we first calculate the intellectual distance associated with each novel journal pair referenced in a publication. Specifically, we compute the complement of the cosine similarity between the two journals, based on their citation co-occurrence patterns with other journals in the preceding three years. The cosine similarity captures the extent to which the two journals appear in similar citation contexts. A high cosine similarity indicates that the journals are frequently cited alongside the same set of other journals, suggesting a well-established relationship and low intellectual distance between them. In contrast, a low similarity score indicates that the journals originate from more distant knowledge areas, implying greater intellectual distance between them.

For each journal in a novel journal pair, we construct a co-citation profile that records the number of publications over the past three years in which the journal has been co-cited with every other journal. We compute the dot product of the two journals’ co-citation profiles to measure the overlap in co-citation patterns. This involves identifying all journals common in the two journals’ co-citation profiles, multiplying their respective publication counts, and summing these products.

We then calculate the cosine similarity between the two journals by scaling this dot product by the Euclidean norms of the two co-citation profiles. The Euclidean norm reflects the overall magnitude of a journal’s co-citation activity—how frequently it is co-cited with other journals. This normalization ensures that the similarity score captures the pattern of co-citation rather than just the volume, thereby indicating how closely related the two journals are in the prior citation landscape.


$$Cosine\;Similarity_{JiJj\;novel}=\frac{Ji\cdot Jj}{\left\|Ji\|\|Jj\right\|}\;$$

where *Ji* and *Jj* represent the co-citation profiles of the journals involved in the novel journal pair Ji-Jj.

Finally, to capture the intellectual distance of each novel recombination, we subtract the cosine similarity from 1. This complement transformation ensures that higher values indicate greater conceptual distance between the journals. A score of 1 for a novel journal pair indicates that the two journals have no prior co-citation overlap, suggesting they come from highly disparate knowledge areas and are difficult to recombine. A score of 0 implies a cosine similarity of 1, indicating the journals are frequently co-cited in similar contexts and, therefore, easy to recombine.


$$Intellectual\;distance_{JiJj\;novel}=1-Cosine\;Similarity_{JiJj\;novel}\;$$

**Fig 1:**
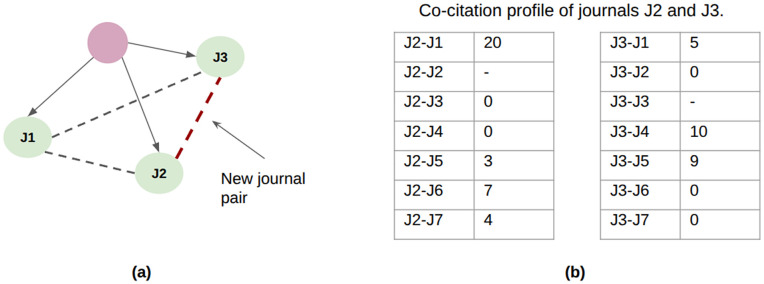
An illustration of a publication’s referenced journal pairs and their co-citation profiles for Novelty measurement. (a) An illustration of journal pairs (J1-J2, J2-J3 and J1-J3) referenced in a publication. (b) An illustration depicting co-citation profiles of journals J2 and J3 in a novel journal pair (J2-J3).

We illustrate this calculation using an example where a publication cites only three journals: J1, J2, and J3 in its references (Fig 1(a)). The resulting journal pairs for this publication are J1–J2, J2–J3, and J1–J3. Suppose that J2–J3 is a novel pair, i.e., the two journals have not been cited together in any publication over the past 20 years. Let us also assume that the universe of journals in our dataset consists of J1, J2, ..., J7. Fig 1(b) displays the co-citation profiles for J2 and J3. For instance, J2 has been co-cited with J1 in 20 publications, with J5 in 3, with J6 in 7, and with J7 in 4, and has not been co-cited in any with any other journals in the past three years. A similar co-citation profile is also created for J3. Among the co-cited journals of J2 and J3, J1 and J5 appear alongside both J2 and J3, making them the shared co-citation links between the two. The resulting dot product of the co-citation profiles of J2 and J3 is 127 (20×5 + 3 ×9), and the corresponding Euclidean norms are 21.77 and 14.35, respectively. This yields a cosine similarity of 0.32, and the intellectual distance between the journals J2 and J3 thus becomes 0.68.

**Novelty breadth** (𝒩oveltyℬreadth )

We evaluate the metric for *novelty breadth* of a publication by adding the intellectual distances between all new journal combinations in the references of the publication.


$$𝒩oveltyℬreadth=\sum_{novel\;JiJj}Intellectual\;distance_{JiJj}\;$$

This metric reflects the cumulative conceptual distance involved in the publication’s knowledge integration. A higher novelty breadth indicates that novel recombinations in publications engage with a broader, more diverse set of knowledge elements.

**Novelty distance** (𝒩ovelty𝒟istance )

We evaluate *novelty distance* of a publication through the intellectual distance between the most disparate novel journal combination.


$$𝒩ovelty𝒟istance=\max_{novel\;JiJj}Intellectual\;distance_{JiJj}\;$$

A higher *novelty distance* reflects that the publication integrates highly disparate ideas.

### Independent variable: Industry publishing contribution to a field

Industry publishing contribution is our key variable of interest. We use the percentage of publications with at least one industry author to indicate industry publishing contribution to a field.

A publication can be assigned to more than one field. This violates the independence assumption in regression models. We address this problem by assigning a single field to each publication, flattening the measure so that each publication has a unique value for the field’s industry publishing contribution. We assign the field corresponding to the ‘maximum’ value of industry publishing contribution across all the fields of a publication as the field of the publication. We also run an additional analysis by assigning the field associated with the ‘minimum’ value of industry publishing contribution among the fields of the publication to the publication to get a lower bound on our estimates for robustness (Table S6.1: SI Appendix S6).

Consequently, the measure of industry publishing contribution to a field, corresponding to a given publication in year *y* takes the following form:


$$\begin{array}{cc} Ind\;PubContribution_{f}=\max_{s}( & \%\text{ publications with at least one}\\ & \text{industry-affiliated author in field }s\\ & \text{ in year }y)\; \end{array}$$

where, *s* is the set of fields the publication belongs to, and the field *f* assigned to a publication is given by,


$$\begin{array}{cc} f=\arg\max_{s}( & \%\text{ publications with at least one industry-affiliated}\\ & \text{ author in field }s\text{ in year }y)\; \end{array}$$

We compute additional field-level metrics for a given publication corresponding to the field *f* in year *y*.

Since research takes time to develop and be published, and our focus is on how industry publishing contributions shapes research within fields, we lag the measure of industry publishing contribution by two years. Additionally, we lag all field-level control variables by two years in our analysis for consistency. We also use four-year lagged field-level variables for robustness (see SI Appendix S7 for details).

We also account for potential confounding factors at both the publication and field levels (see SI Appendix S3 for details). At the field level, we control for the field’s size and appliedness. At the publication level, we include controls for various characteristics such as the number of authors, the number of references cited, the number of fields in which the publication is classified, whether the publication involves international collaboration, the number of organizations affiliated with the authors, and the institutional affiliations of the authors. A brief description of variables is provided in Table 2.

**Table 2:** Description of variables.

Variable name	Description
**Publication-level**	
*T*_*size*_	Number of authors
*N*_*refs*_	Number of references
*Pub*_*fields*_	Number of fields a paper belongs to
*International*	Dummy with value 0 if all the authors of a publication are from the same country and 1 otherwise
*N*_*orgs*_	Number of organizations of paper authors
*JIF*	Journal impact factor
*Industry*	Dummy with value 1 if all coauthors are affiliated with industry and 0 otherwise
*UI*	Dummy with value 1 if at least one coauthor affiliated with university and at least one coauthor affiliated with industry and 0 otherwise
𝒩ovelty𝒪ccurrence	Dummy with value 1 if the publication references a novel journal pair and 0 otherwise
𝒩oveltyℬreadth	*Novelty breadth* of publication
𝒩ovelty𝒟istance	*Novelty distance* of publication
**Field-year-level**	
$Ind\;PubContribution_{f}\;$	Industry publishing contribution to a field
*Field*_*size*_	Number of publications in a field
*Appliedness*_*f*_	Appliedness of a field

## Results

**Fig 2:**
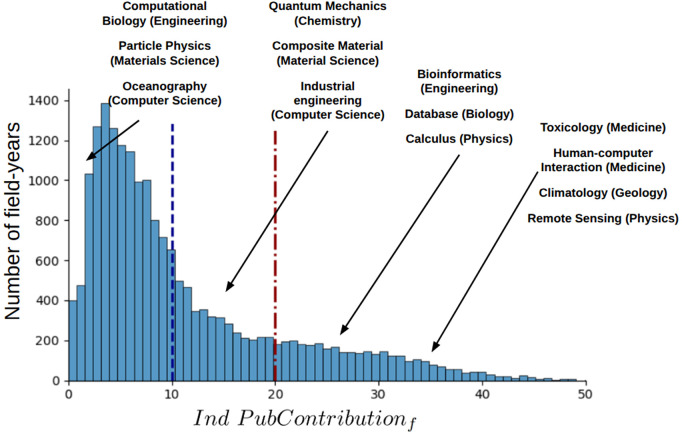
Distribution of the industry publishing contribution across field-years. The blue dashed vertical line shows the median industry publishing contribution across field-years (9.99%), and the red dot-dashed vertical line represents the median industry publishing contribution for novel papers across field-years (19.27%).

Fig 2 illustrates the distribution of industry publishing contribution across field-years. The median industry publishing contribution to a field is 9.99%, reaffirming university’s role as the dominant source of scientific output in most field-years. However, industry publishing contribution is far from negligible; in several field-years, industry accounts for as many as 40% of the publications. Selected field names from the year 2012 are included in the figure to exemplify the positioning of different fields within the distribution. Fields such as Oceanography in Computer Science and Particle Physics in Material Science exhibited less than 2% industry publishing contribution in 2012, while fields like Artificial Intelligence in Computer Science and Composite Materials in Materials Science showed around 15% industry contribution. In contrast, domains such as Database in Biology (25%) and Remote Sensing in Physics (35%) occupied the higher end of industry publishing involvement.

This variation in industry publishing contribution highlights that industry plays a prominent publishing role in specific fields. Notably, the median industry publishing contribution to field-years rises to 19.27% for only novel publications (𝒩ovelty𝒪ccurrence = 1 ), in line with our expectation that high industry contribution to a field is associated with producing novel scientific work.

Our analysis focuses on two dimensions: the propensity of a publication to exhibit novelty (*novelty occurrence*) and the degree of novelty captured through *novelty breadth* and *novelty distance*. As expected, only a small proportion of publications, 446,637 out of 11,128,665 (4%), are classified as novel, highlighting the inherent rarity of *novelty occurrence*. However, the substantial absolute number of novel publications provides a strong empirical setting to systematically examine variations in the degree of novelty (*novelty breadth* and *novelty distance*).

Importantly, because *novelty occurrence* is rare, analyzing the degree of novelty across the full sample may be confounded by the underlying likelihood of novelty occurrence itself. This distinction is especially important in our field-level analysis, where aggregate patterns may mask whether the observed effects are driven by the frequency of novelty occurrences or by the degree of novelty. This underscores the need to conduct separate empirical analyses of *novelty occurrence* and the degree of novelty to interpret field-level dynamics accurately.

Therefore, we adopt a two-part empirical strategy. We use Logistic regression to estimate the relationship between industry publishing contribution to a field and the likelihood of that a publication being is novel within that field. Although novel publications represent only 4% of the sample, the large sample size ensures consistent estimates from logistic regression [83]. Since *novelty breadth* and *novelty distance* are skewed (Table S3.1, SI Appendix S3), we employ OLS regression using log-transformed measures of novelty (*novelty breadth* and *novelty distance*) to assess how industry publishing contribution to a field relate to the extent of novelty in publications within the field. We use publication-year fixed effects and robust standard errors clustered at the field level for all regression estimates. Given the substantial size of our sample, we employ the Bayesian Information Criterion (BIC) and Akaike Information Criterion (AIC) to assess and compare the goodness of fit among the regression models [84].

### Novelty occurrence (*novelty occurrence*)

Models 1–2 in Table 3 present the Logistic regression estimates for the relationship between industry publishing contribution to a field and *novelty occurrence*. In model 1, we begin by including the control variables. The findings for the control variables indicate that in line with prior work, the likelihood of *novelty occurrence* increases with team size (*T*_size_) highlighting the importance of teams in driving novel recombinations [85], and decreases when the publication involves international collaboration [86] or a collaboration between authors from several organizations (*N*_*orgs*_). In addition, a higher number of references in a publication (*N*_*refs*_) is positively associated with *novelty occurrence*, potentially reflecting a greater scope for knowledge integration. We also find a positive relationship between the number of fields associated with a publication (*Pub*_*fields*_) and *novelty occurrence*, suggesting that interdisciplinary works may increase the chances of new recombination. A higher journal impact factor (*JIF*) is associated with a lower likelihood of novelty occurrence, in line with prior work [2]. Further, on average, publications with some authors affiliated with university and some with industry (*UI*) have higher odds for *novelty occurrence* than those authored by solely university- or solely industry-affiliated authors (This reflects a higher potential of industry to introduce new ideas within a field.).

**Table 3:** Regression estimates for the relationship between industry publishing contribution and different aspects of novelty.

	(1)	(2)	(3)	(4)	(5)	(6)	(7)	(8)	(9)
	Logit(𝒩ovelty𝒪ccurrence)			Ln(𝒩oveltyℬreadth)			Ln(𝒩ovelty𝒟istance)		
*Ln*(*T*_*size*_)	0.092***	0.044***	0.039***	−0.007***	−0.010***	−0.011***	−0.010***	−0.008***	−0.008***
	(0.015)	(0.011)	(0.011)	(0.003)	(0.003)	(0.003)	(0.001)	(0.001)	(0.001)
*Ln*(*Field*_*size*_)	-0.459***	−0.662***	−0.664***	−0.141***	−0.151***	−0.150***	−0.026***	−0.020***	−0.021***
	(0.164)	(0.113)	(0.111)	(0.017)	(0.015)	(0.015)	(0.005)	(0.005)	(0.005)
*Ln*(*N*_*refs*_)	0.469	0.089	0.044	1.215***	1.203***	1.202***	0.050	0.057*	0.058**
	(0.354)	(0.273)	(0.275)	(0.100)	(0.095)	(0.095)	(0.033)	(0.030)	(0.029)
*Ln*(*Pub*_*fields*_)	0.039**	−0.003	−0.004	−0.011***	−0.017***	−0.017***	−0.003	0.000	0.000
	(0.017)	(0.014)	(0.013)	(0.004)	(0.004)	(0.004)	(0.002)	(0.002)	(0.002)
*International*	−0.144***	−0.078***	−0.082***	−0.011***	−0.005	−0.006	0.004***	0.000	0.000
	(0.013)	(0.011)	(0.010)	(0.004)	(0.004)	(0.004)	(0.001)	(0.001)	(0.001)
$Ln(N_{orgs})\;$	-0.027**	−0.003	0.010	−0.008***	−0.006***	−0.005**	0.003***	0.002*	0.001
	(0.014)	(0.010)	(0.010)	(0.002)	(0.002)	(0.002)	(0.001)	(0.001)	(0.001)
*Ln*(*JIF*)	−0.007	−0.048	−0.063	−0.014**	−0.012**	−0.013**	−0.014***	−0.015***	−0.015***
	(0.049)	(0.040)	(0.041)	(0.005)	(0.005)	(0.005)	(0.002)	(0.002)	(0.002)
*UI*	0.379***	0.045**	0.192***	0.035***	0.005	0.017***	−0.022***	−0.004**	−0.012***
	(0.045)	(0.021)	(0.026)	(0.007)	(0.006)	(0.006)	(0.004)	(0.002)	(0.003)
*Industry*	0.284***	−0.153***	0.264***	0.028***	−0.010	0.022**	−0.022***	0.001	−0.026***
	(0.060)	(0.027)	(0.041)	(0.010)	(0.009)	(0.009)	(0.005)	(0.003)	(0.005)
*Appliedness*_*f*_	-0.177**	−0.244***	−0.268***	0.722***	0.719***	0.718***	0.027	0.029	0.030
	(0.089)	(0.075)	(0.076)	(0.086)	(0.082)	(0.082)	(0.032)	(0.028)	(0.027)
$Ind\;PubContribution_{f}\;$		0.384***	0.462***		0.044***	0.050***		−0.026***	−0.031***
		(0.045)	(0.044)		(0.008)	(0.008)		(0.005)	(0.005)
*UI*×*Ind* *PubContribution*_*f*_			-0.275***			−0.024***			0.016***
			(0.020)			(0.006)			(0.004)
*Industry*×*Ind* *PubContribution*_*f*_			-0.439***			−0.037***			0.030***
			(0.032)			(0.009)			(0.006)
*IMR*	−29.348***	−37.736***	−38.611***	31.591***	31.259***	31.250***	1.813	2.015*	2.019*
	(8.245)	(6.296)	(6.335)	(3.675)	(3.502)	(3.506)	(1.242)	(1.134)	(1.093)
Constant	9.826**	13.914***	14.354***	−17.005***	−16.827***	−16.822***	−1.157*	−1.265**	−1.268**
	(3.926)	(3.006)	(3.027)	(1.965)	(1.873)	(1.875)	(0.664)	(0.606)	(0.584)
Year FE	Yes	Yes	Yes	Yes	Yes	Yes	Yes	Yes	Yes
Observations	11,128,665	11,128,665	11,128,665	446,637	446,637	446,637	446,637	446,637	446,637
Groups	1639	1639	1639	1384	1384	1384	1384	1384	1384
AIC	3082295	3044583	3038086	1050241	1049388	1049310	125030	122523	122165
BIC	3082665	3044968	3038499	1050373	1049531	1049475	125163	122666	122330

Standard errors in parentheses.

* *p* < 0.10, ** *p* < 0.05, *** *p* < 0.01.

All independent variables except dummy variables are standardized about the mean.

IMR: Inverse-Mill’s Ratio. Year FE: Year Fixed-effects.

We add our key variable of interest (*IndPubContribution*_*f*_) in model 2. Our findings support H1 by revealing a positive relationship between industry publishing contribution to a field and the odds of publications within the field being novel (Between models 1 and 2, the AIC decreases from 3082295 to 3044583 (>10), and the BIC decreases from 3082665 to 3044968 (>10), indicating a better fit of model 2 over 1. The sign reversal of the *Industry* coefficient between Models 1 and 2 reflects industry’s systematic selection into specific fields, which is expected given that industry-authored publications are more likely to occur in fields with high industry publishing contribution.). Specifically, a one standard deviation increase in the share of industry-authored publications within a field is associated with a 46.8% increase in the odds of a publication being novel.

### Degree of Novelty (*novelty breadth* and *novelty distance*)

Next, we examine how industry contribution to publishing relates to the degree of novelty, focusing on novel publications within the fields. The estimates for control variables in models 4 (for *novelty breadth*) and 7 (for *novelty distance*) (Table 3) are largely consistent with those observed for *novelty occurrence*, with the exception of publication affiliation. On average, solely university-authored publications exhibit greater novelty distance than those from industry, indicating recombination of disparate ideas by university. No significant difference is observed between the two for novelty breadth.

We expect the data to reveal the direction of the relationship between industry publishing contribution and *novelty breadth,* and a negative relationship with *novelty distance*. However, our main results in models 5 and 8 (Table 3) uncover a nuanced trade-off: industry publishing contribution within a field is associated with an increase in *novelty breadth* but a decrease in *novelty distance* of publications within the field (Between models 4 and 5, the AIC decreases from 1050241 to 1049388 (>10), and the BIC decreases from 1050373 to 1049531 (>10), indicating a better fit of model 5 over 4. Between models 7 and 8, the AIC decreases from 125030 to 122523 (>10), and the BIC decreases from 125163 to 122666 (>10), indicating a better fit of model 8 over 7.). The findings align with H2a and H3. In terms of the effect size, a one standard deviation increase in the industry publishing contribution to a field is associated with a 4.4% rise in *novelty breadth* and a 2.6% decline in *novelty distance* of publications within the field.

A larger effect size for *novelty occurrence* suggests higher odds of producing novel research within a field. In contrast, the degree of novelty—captured by *novelty breadth* and *novelty distance*—shows more modest effects. It is important to note that, while the occurrence of novelty is relatively rare, achieving a high degree of novelty is even rarer. Therefore, even modest effect sizes for *novelty breadth* and *novelty distance* are meaningful and substantively important.

Our main results suggest that while greater industry publishing in a field can foster novel recombinations by expanding the idea pool, introducing new perspectives, and promoting the integration of diverse knowledge to address complex, industry-relevant problems, it may simultaneously divert academic attention away from exploratory, blue-sky research that depends on combining conceptually distant knowledge.

It is worth noting that our findings for all three measures, novelty occurrence, novelty breadth, and novelty distance, hold even after controlling for the degree of appliedness of a field. Appliedness of a field refers to the extent of commercial application of research within that field (see SI Appendix S3.1). While appliedness of a field captures the problem orientation of research, industry publishing contribution reflects the participation of firms as knowledge producers within the scientific field. These two dimensions, therefore, capture distinct aspects of the research environment. An explanation for the persistence of our results after accounting for appliedness is that, in fields with low appliedness, science can provide a theoretical understanding of the technological components and their interactions [23]. This suggests that the observed relationship between industry publishing contribution and novelty is not simply driven by the applied nature of the field, but also reflects the role of industry actors participating in scientific knowledge production.

### University vs industry: Which entity drives novelty?

Having analyzed the association between industry publishing contribution and novelty, an important question that remains is whether the observed patterns are predominantly driven by university or industry-authored publications. If the observed relationship between industry publishing contribution and novelty is driven primarily by industry-authored publications, its effect would be limited to industry outputs. However, if the relationship persists across the entire field—including university-authored publications—it would suggest that industry publishing activity plays a formative role in shaping the field’s overall novelty. We explore this by comparing the relationship of novelty across the three groups of affiliations: solely university-authored (all authors affiliated with university), UI-authored (with some authors affiliated with university and some with industry), and solely industry-authored (all authors affiliated with industry) publications.

The negative coefficient estimates for the interaction of industry publishing contribution with UI- and solely industry-authored publications in model 3 (Table 3) reveal that the relationship between industry publishing contribution and *novelty occurrence* within a field is the strongest for solely university-authored publications. This suggests that university-authored publications are the primary drivers of the positive relationship between industry publishing contribution and *novelty occurrence* within fields. The effect sizes indicate that a one standard deviation increase in industry publishing contribution to a field is associated with a 58.7% increase in the odds of a solely university-authored publication exhibiting *novelty occurrence*, while the odds increase by 20.6% for UI-authored publications, and by only 2.3% for solely industry-authored publications.

Similar to *novelty occurrence*, the findings in model 6 (Table 3) indicate that university contributes the most to the positive relationship between industry publishing contribution and *novelty breadth* within fields. A one standard deviation increase in industry publishing contribution to a field corresponds to a 5% increase in *novelty breadth* for solely university-authored publications, a 2.6% increase for UI-authored, and a 1.3% increase for solely industry-authored publications.

However, Model 9 (Table 3) reveals that solely university-authored publications also exhibit the largest reduction in *novelty distance* as industry publishing contribution increases. This pattern suggests that university plays a central role in driving the negative relationship between industry publishing contribution and *novelty distance*. In terms of effect size, a one standard deviation increase in industry publishing contribution to a field is associated with a 3.1% decrease in *novelty distance* for solely university-authored publications, a 1.5% decrease for UI-authored, and a 0.1% decrease for solely industry-authored publications. Additionally, we also observe a cross-over pattern in the interaction effects: university-authored publications exhibit lower *novelty occurrence* and *novelty breadth* compared to other groups in fields with less than ~25% industry publishing contribution but higher novelty breadth in fields with more than ~25% industry publishing contribution (largely Bio-medical research related fields).

Therefore, the relationship between industry publishing contribution and all three measures of novelty is not exclusively driven by industry-authored publications, and university is an important driver of the relationship. These results highlight the importance of greater industry publishing contribution in driving the novelty of entire fields.

### Institution research ranking

We additionally examine whether the relationship between industry publishing contribution and the three novelty dimensions differs for publications affiliated with high-ranking compared to other research institutions. To do so, we classify a publication as affiliated with a top-500 institution (*Top* 500) if at least one author’s institutional affiliation appears in the top-500 of the SCImago Institutions Rankings: Research Indicator [87].

Table 4 shows that high-ranking institutions are, on average, associated with lower *novelty occurrence* (Model 1) and *novelty breadth* (Model 3), but with higher *novelty distance* (Model 5). This pattern suggests that high-ranking institutions tend to recombine knowledge components that are cognitively distant from one another.

**Table 4:** Regression estimates of the relationship between industry publishing contribution and novelty dimensions across top-500 (*Top* 500) and other research institutions.

	(1)	(2)	(3)	(4)	(5)	(6)
	Logit(𝒩ovelty𝒪ccurrence)		Ln(𝒩oveltyℬreadth)		Ln(𝒩ovelty𝒟istance)	
*Ln*(*T*_*size*_)	0.048***	0.048***	−0.011***	−0.011***	−0.004***	−0.004***
	(0.012)	(0.012)	(0.002)	(0.001)	(0.001)	(0.001)
*Ln*(*Field*_*size*_)	-0.270***	−0.270***	−0.176***	−0.176***	−0.025**	−0.025**
	(0.052)	(0.052)	(0.032)	(0.012)	(0.010)	(0.010)
*Ln*(*N*_*refs*_)	1.385***	1.386***	1.311***	1.311***	0.070***	0.070***
	(0.037)	(0.037)	(0.103)	(0.028)	(0.022)	(0.022)
*Ln*(*Pub*_*fields*_)	0.012	0.012	−0.023***	−0.023***	0.000	0.000
	(0.012)	(0.012)	(0.003)	(0.001)	(0.001)	(0.001)
*International*	−0.075***	−0.075***	−0.001	−0.001	0.001	0.001
	(0.011)	(0.010)	(0.004)	(0.004)	(0.001)	(0.001)
*Ln*(*N*_*orgs*_)	0.002	0.002	−0.001	−0.001	0.000	0.000
	(0.010)	(0.010)	(0.002)	(0.002)	(0.001)	(0.001)
*Ln*(*JIF*)	0.113***	0.113***	−0.006	−0.006***	−0.003***	−0.003***
	(0.028)	(0.028)	(0.004)	(0.002)	(0.001)	(0.001)
*UI*	0.043**	0.041**	0.003	0.003	−0.004***	−0.005***
	(0.021)	(0.020)	(0.006)	(0.004)	(0.002)	(0.002)
*Industry*	−0.162***	−0.158***	−0.011	−0.012***	−0.004*	−0.003
	(0.027)	(0.026)	(0.008)	(0.004)	(0.002)	(0.002)
*Appliedness*_*f*_	0.069**	0.069**	0.839***	0.839***	0.052***	0.053***
	(0.032)	(0.032)	(0.090)	(0.024)	(0.019)	(0.019)
$Ind\;PubContribution_{f}\;$	0.383***	0.370***	0.100***	0.100***	−0.030***	−0.032***
	(0.045)	(0.048)	(0.027)	(0.013)	(0.009)	(0.009)
*Top* 500	−0.059***	−0.065***	−0.015***	−0.015***	0.003*	0.002
	(0.011)	(0.012)	(0.004)	(0.003)	(0.002)	(0.001)
*Top* 500×*Ind* *PubContribution*_*f*_		0.022		−0.001		0.003**
		(0.016)		(0.003)		(0.002)
*IMR*	−7.484***	−7.473***	35.172***	35.170***	2.112***	2.123***
	(0.726)	(0.721)	(3.773)	(1.041)	(0.802)	(0.803)
Constant	−0.457	−0.459	−18.940***	−18.939***	−1.339***	−1.344***
	(0.349)	(0.347)	(2.016)	(0.557)	(0.429)	(0.430)
Year FE	Yes	Yes	Yes	Yes	Yes	Yes
Observations	11,128,665	11,128,665	446,637	446,637	446,637	446,637
Groups	1639	1639	1639	1236	1236	1236
AIC	3046619	3046576	1043734	1043738	110574	110562
BIC	3046818	3046789	1043877	1043903	110717	110716

Standard errors in parentheses.

* *p* < 0.10, ** *p* < 0.05, *** *p* < 0.01.

All independent variables except dummy variables are standardized about the mean.

IMR: Inverse-Mill’s Ratio. Year FE: Year Fixed-effects.

The interaction term *Top* 500×*Ind* *PubContribution*_*f*_ is not statistically significant in the models for *novelty occurrence* (Model 2) or *novelty breadth* (Model 4), suggesting that the relationship between industry publishing contribution and these novelty dimensions is similar for publications from top-ranked and other institutions. In contrast, the positive coefficient for the interaction *Top* 500×*Ind* *PubContribution*_*f*_ in Model 6 suggests that the negative relationship between industry publishing contribution and *novelty distance* is less pronounced for publications by high-ranking institutions compared to other publications.

The effect sizes indicate that, for publications from top-ranked institutions, a one standard deviation increase in the share of industry-authored publications within a field is associated with a 2.9% decline in *novelty distance*, compared to a 3.2% decline for other publications within the same field.

Therefore, while the associations between industry publishing contribution and both *novelty occurrence* and *novelty breadth* are similar across top-ranked research universities, the negative association with *novelty distance* is less pronounced for publications from top-ranked research universities. This suggests that high-ranking institutions are able to capture the benefits of increased *novelty occurrence* and *novelty breadth*, while partially mitigating the decline in *novelty distance*. One possible explanation is that top-ranked institutions possess stronger research infrastructure, greater autonomy in agenda-setting, and more stable access to long-term funding, which may allow researchers to pursue more exploratory and conceptually distant lines of inquiry, even in fields with substantial industry publishing. These findings remain consistent when the comparison is conducted using the top 100 research institutions versus others (SI Appendix S12).

We conduct additional analysis by stratifying the operationalization of industry publishing contribution to a field into two parts: industry publishing contribution emanating from the percentage of publications authored by university and industry scientists together within the field, and that emanating solely from industry-authored publications (see SI Appendix S5 for details). The findings (Table S5.1, SI Appendix S5) remain largely consistent with the main results. UI publishing contribution is positively associated with *novelty occurrence* and negatively associated with *novelty distance*, although the association with *novelty breadth* is not statistically significant. Similarly, sole-industry publishing contribution is positively associated with both *novelty occurrence* and *novelty breadth*, while its association with *novelty distance* is not statistically significant. These variations in statistical significance in some of the robustness tests may stem from the field-level nature of our analysis, where limited within-field variation could affect the precision of some estimates.

We also conduct several robustness checks for our main results. Our estimates remain consistent across alternative specifications for industry publishing contribution (SI Appendix S6 and S11), lag of field-level variables (SI Appendix S7), field assignment (SI Appendix S8), author affiliation (SI Appendix S9), and novelty (SI Appendix S10).

## Discussion

Publishing novel research is crucial for scientific advances [3,88] and technological breakthroughs [1]. Our study examines a field-level driver of novelty in science—the extent of industry contribution to publishing within a scientific field. By analyzing a longitudinal dataset of 11.1 million publications spanning 1,639 STEM fields published over 15 years between 2000 and 2014, our findings uncover how industry publishing contribution to a field shapes scientific novelty across fields, distinguishing between *novelty occurrence* and the degree of novelty, captured through *novelty breadth* and *novelty distance*.

Our findings reveal that greater industry publishing contribution to a field is associated with increased odds for *novelty occurrence* and increased *novelty breadth*, but diminished *novelty distance*. The findings suggest that a high extent of industry publishing within a field can foster novel recombinations by expanding the idea pool and introducing new perspectives within a field, and promote the integration of diverse novel recombinations of knowledge that address complex, industry-relevant problems. At the same time, it may divert academic attention away from more exploratory, blue-sky curiosity-driven research that combines conceptually distant knowledge. These results underscore the nuanced role of industry publishing in shaping distinct dimensions of novelty across scientific fields. Additionally, we find that university is an important driver of our results across the three dimensions of novelty. This reinforces our argument that industry publishing contribution to a field has implications for the entire field.

We also report that with heightened industry publishing activity in a field, both top and lower-ranked research institutions experience similar gains in *novelty occurrence* and *novelty breadth*. However, high-ranked research institutions experience a lower decline in *novelty distance* as industry publishing in a field increases. These findings suggest that leading research institutions may be better equipped to sustain more exploratory and conceptually distant research trajectories, even in fields characterized by substantial industry publishing. Such institutions may also be able to accommodate applied, industry-oriented research alongside longer-horizon, curiosity-driven inquiry, rather than allowing the former to crowd out the latter. One plausible mechanism is that top-ranked universities benefit from stronger research infrastructure, greater discretion in setting research agendas, and more stable access to long-term funding, which together may enable researchers to pursue riskier and more distant recombinations of knowledge despite increasing industry involvement.

These findings also have implications for science policy. Policymakers worldwide continue to promote university-industry collaboration as a mechanism for fostering innovation and enhancing the societal relevance of scientific research. However, debates persist over the consequences of such engagement for the trajectory of scientific fields and the nature of the knowledge produced. On the one hand, a high degree of industry publishing in a field can help develop industry-ready science and technology [89,90]. Conversely, there is a rising concern around too much industry involvement in research and industry driving the academic research agenda [91–93]. Our work contributes to this conversation by offering nuanced implications for scientific novelty.

Our work informs policymakers that promoting industry participation in scientific publishing can serve as an effective lever to stimulate novelty within fields through increased *novelty occurrence*, and foster broad knowledge integration (*novelty breadth*) conducive to complex problem-solving. However, encouraging industry publishing also entails a trade-off: decreased *novelty distance*, which may stagnate long-term scientific progress within the field. If policymakers seek to foster more exploratory and blue-sky research in fields with high industry publishing contribution, complementary mechanisms may be necessary to incentivize researchers to engage in more distant, high-risk recombinatory activities in those fields. For example, policies could fund long-term curiosity-driven research programs or create institutional buffers that protect academic researchers from short-term performance pressures in such fields.

Additionally, our results indicate that the negative association of increased industry publishing with *novelty distance* is attenuated in top-ranked research universities. Policies that encourage industry engagement within research-intensive institutions, or that strengthen the capacity of other institutions to support long-horizon, curiosity-driven research, may help preserve more exploratory forms of novelty, while retaining the benefits of industry participation on other novelty dimensions.

More broadly, our findings emphasize that industry participation in scientific publishing has a multifaceted influence on scientific progress, shaping entire fields. This also underscores the importance for policymakers to monitor and account for field-level metrics, such as the extent of industry publishing contribution to a field, when designing university-industry engagement policies.

While we have incorporated several controls and lagged field-level variables, our work does not offer causal inference. Future research could explore the precise mechanisms underlying our results. In addition, further work could examine how the relationship between novelty and the scientific impact of publications varies across fields. Novelty in scientific research is inherently multidimensional, and no single measurement approach fully captures its different aspects [40]. Prior studies have also highlighted that relatively few works systematically examine the multifaceted nature of recombinant novelty [40,42,43]. While we adopted a pairwise combinatorial novelty approach [2,94] and captured the aspects of *novelty occurrence* and degree of novelty through *novelty breadth* and *novelty distance*, other approaches conceptualize and operationalize novelty differently. For example, some studies conceptualize novelty through semantic representations of scientific texts using natural language processing methods [95], others through network-based measures reflecting the structural position of recombined knowledge [45,48], or through impact-based disruption measures [96]. Future work could extend our analysis to other operationalizations and conceptualizations of novelty. Additionally, our analysis focuses on how industry participation in publishing shapes novelty within fields. Future research could extend this work by examining university and industry contributions across both publications and patents to understand how different disclosure channels influence knowledge novelty.

## Supporting information

SI Appendix UIC Novelty Appendix Plos R2. (PDF)
