# Supplementary material for: The field factor: Industry publishing contribution and novelty in science
Source: PLoS One. 2026 Apr 27;21(4):e0346227. doi: 10.1371/journal.pone.0346227 (PMC13120708; doi:10.1371/journal.pone.0346227)
Supplement: SI Appendix — (PDF) [file pone.0346227.s001.pdf]

SI Appendix

S1 Field classification

We use a combination of Level 0 and Level 1 concept codes provided by OpenAlex to represent a field [1, 2]. Since Level 1 concepts are embedded within Level 0 concepts, we operationalize a field as the intersection of a Level 0 and a Level 1 concept. For example, combining the Level 0 concept of Biology with the Level 1 concept of Zoology yields the field Zoology in Biology. Similarly, combining the Level 0 concept of Chemistry with the Level 1 concept of Quantum Mechanics yields the field Quantum Mechanics in Chemistry.

Importantly, we do not use Level 1 concepts alone because the same Level 1 concept can correspond to substantively different research communities depending on the broader disciplinary context. This intersection-based definition provides a more meaningful approximation of a scientific community whose members work on related problems and employ similar methodological approaches. For instance, a publication assigned the Level 1 concept Machine Learning within the Level 0 concept Chemistry is likely using machine learning as a tool within a chemical research context, whereas a publication assigned Machine Learning within Computer Science is more likely to develop or advance machine learning methods themselves. Although both publications share a Level 1 label, they typically engage with different scholarly communities and norms.

OpenAlex uses a deep learning-based approach for the concept assignment and provides confidence scores for each Level 0 and Level 1 concept assignment. If the confidence score for a publication's Level 0 or Level 1 concept is above the median level of the confidence scores across all publications, we assign the concept to the publication. The cut-off confidence score is 0.3 for Level 0 concepts and 0.26 for Level 1 concepts.

Since the field operationalization depends on these concept assignments, and the concept assignments vary in their confidence levels, field classification may be sensitive to the confidence thresholds used. To address this concern, we conduct robustness checks (*SI Appendix* S8) using an alternative more stringent confidence threshold for field assignment and confirm that our main results remain qualitatively unchanged.

S2 Author affiliation classification

We define a university as an institution whose primary objective is education and/or advancing science [3]. OpenAlex database classifies affiliation type for each author into the following: 'education', 'facility', 'company', 'healthcare', 'archive', 'nonprofit', 'government' and 'other'.

Since the focus of this paper is on industry's publishing contribution to science dynamics, we drop publications with at least one author classified as 'government' or 'other'.[1]

In accordance with our definition of a university, we classify the category of 'education' as university. The category of 'facility' includes research facilities and we classify the same as university.

We classify organizations in the categories of 'company' as industry. The primary goal of 'healthcare' organizations is patient care, with research and education as secondary goals. Therefore, we classify these organizations as industry. The category 'archive' consists of organizations which hold archives like libraries and museums. Therefore, we also classify the category 'archive' as industry.

In summary, we classify authors from with the categories of 'education' and 'facility' as university-affiliated authors, and authors from with the categories of 'company', 'healthcare' and 'archive' as industry-affiliated authors. For robustness of our findings, we also use an alternate broader classification for university, where we include authors affiliated with the category 'nonprofit' as university authors (see section S9).

[1]We include these publications in the in the calculation for a field's size.

S3 Control variables

S3.1 Field-level controls

$Field_{size}$

We control for the size of the field f of a publication in year y by,

$$Field_{size} = \#\text{ publications in field } f \text{ in year } y$$

$Appliedness_f$

Prior work posits that industry publishing contribution is higher in more applied fields [4, 5, 6]. Research in applied fields involves problem-solving and developing technologies, which could encourage novel recombinations. We account for this by controlling for the degree of appliedness of a field. A good proxy for the degree of appliedness of a field can be found by assessing how frequently its publications are cited in patents. Our measure for a field's appliedness follows the approach outlined by Bikard et al. (2019) [7]. To operationalize this, we calculate the number of times publications from a specific field in year y are cited in patents over the next five years (between year y and y+5) relative to the size of the field in that year. We then apply a logarithmic transformation to obtain the degree of appliedness of the field in year y.

$$Appliedness_f = Ln(1 + \frac{\#\text{Citations to patents between years y to y+5}}{Field_{size}})$$

S3.2 Publication-level controls

Our independent variables are at the level of the field. The industry publishing contribution to a field could have a systematic association with variables at the publication level. We control for publication-level variables that can confound the relationship between the field-level independent variables and the novelty of publications in the field.

T_{size}

Some fields can have a larger coauthor team size than others [8][2] and team size has an inverted-U-shaped relationship with the novelty of publications [9] which can bias our estimates. Therefore, we control the number of coauthors of a publication.

N_{refs}

There can be variation between fields in the tendency to cite more or less references in the publications. A higher number of references could be associated with more journal recombinations and higher novelty. We account for this by controlling the number of references in a publication.

Pub_{fields}

Publications in interdisciplinary fields are more likely to be classified in more fields. Publications with interdisciplinary teams are associated with a higher degree of novelty [9], which can bias our estimates. Therefore, we control for the number of fields a publication is associated with.

[2]In our data, the median industry publishing contribution to the field level is lower than that observed at the paper-field level. This indicates that fields where the industry publishes more are generally larger. A similar pattern is evident when examining the UI publishing contribution and the sole-industry publishing contribution to a field

International

Some research fields align themselves better for international collaboration of authors [10, 11]. International collaboration can also impact the novelty of the publications [12]. Therefore, we also control whether the coauthor team members are from different countries.

N_{orgs}

The coauthors of a publication can belong to the same or distinct organizations. Some fields could be more aligned for inter-organizational collaboration than others [13]. A higher count of organizations of a publication's authors could bring together broader aspects of knowledge, which can impact novelty positively but could also lead to coordination problems, that can impact novelty negatively [14]. We account for this by controlling for the number of authors' organizations in a publication.

JIF

The novelty of a publication may also depend on the journal in which it is published, as prior work shows that highly novel research is more likely to appear in lower impact factor journals [15]. To account for this, we control for the journal impact factor of the publication's journal in the year of publication.

Affiliation (U, I and UI)

We also control for the affiliation of the coauthors of a publication — publications with all authors affiliated with industry (I), publications with some authors affiliated with industry and some with university (UI) and publications where all authors are affiliated with university (U).

Affiliation information is unavailable for some authors in our dataset. If we do not account for the affiliations of all coauthors when classifying a publication as university, industry, or university–industry (UI), it may lead to misclassification. For example, consider a publication with two authors: one affiliated with a university and the other with industry. If the affiliation for the industry author is missing, the publication could be incorrectly classified as a university publication. Such misclassification can introduce bias into our estimates. To mitigate this issue, we exclude publications where affiliation information is missing for at least one coauthor. To correct for potential sample selection bias arising from this exclusion, we apply Heckman's correction [16].

Table S3.1 shows summary statistics for the complete sample and the subsample of novel publications. Tables S3.2 and S9.1 present bivariate correlations between the variables for all publications in the sample and only novel publications respectively.

	Full Sample				Novel publications ($\mathcal{N}ovelty\mathcal{O}ccurrence = 1$)			
	mean	sd	min	max	mean	sd	min	max
$\mathcal{N}ovelty\mathcal{O}ccurrence$	0.04	0.196	0	1				
$\mathcal{N}ovelty\mathcal{B}readth$	0.085	1.204	0	1425.311	2.124	5.642	0.034	1425.311
$\mathcal{N}ovelty\mathcal{D}istance$	0.034	0.169	0	1	0.84	0.188	0.034	1
$Ln(T_{size})$	1.198	0.642	0	6.315	1.312	0.652	0	4.949
$Ln(Field_{size})$	9.467	1.273	3.219	12.107	9.641	1.299	3.219	12.107
$Ln(N_{refs})$	2.933	0.979	0	8.253	3.902	0.673	0.693	8.253
$Ln(Pub_{fields})$	0.983	0.744	0	6.461	1.064	0.778	0	6.174
$International$	0.155	0.362	0	1	0.18	0.385	0	1
$Ln(N_{orgs})$	0.334	0.456	0	4.159	0.399	0.491	0	4.159
$Ln(JIF)$	0.0683	1.150	-6.912	4.994	0.683	0.762	-6.049	4.994
UI	0.106	0.308	0	1	0.156	0.363	0	1
$Industry$	0.11	0.313	0	1	0.111	0.314	0	1
$Appliedness_f$	0.08	0.089	0	1.455	0.097	0.089	0	1.455
$Ind\ PubContribution_f$	16.361	13.386	0	65.714	20.928	12.353	0	59.259
Observations	11,128,665				446,637			

Table S3.1: Summary statistics

	(1)	(2)	(3)	(4)	(5)	(6)	(7)	(8)	(9)	(10)	(11)	(12)
(1) $NoveltyOccurrence$	1.000											
(2) $Ln(T_{size})$	0.036***	1.000										
(3) $Ln(Field_{size})$	0.028***	0.210***	1.000									
(4) $Ln(N_{refs})$	0.202***	0.160***	0.127***	1.000								
(5) $Ln(Pub_{fields})$	0.022***	0.002***	-0.119***	0.043***	1.000							
(6) $International$	0.014***	0.212***	-0.010***	0.110***	0.010***	1.000						
(7) $Ln(N_{orgs})$	0.029***	0.431***	0.038***	0.151***	0.011***	0.565***	1.000					
(8) $Ln(JIF)$	0.109***	0.197***	0.145***	0.547***	0.032***	0.096***	0.142***	1.000				
(9) UI	0.033***	0.257***	0.076***	0.063***	0.010***	0.160***	0.472***	0.100***	1.000			
(10) $Industry$	0.001	-0.014***	0.107***	-0.076***	-0.023***	-0.111***	-0.156***	0.028***	-0.121***	1.000		
(11) $Appliedness_f$	0.039***	0.172***	0.243***	0.067***	0.007***	-0.006***	-0.000	0.126***	0.026***	-0.027***	1.000	
(12) $Ind\ PubContribution_f$	0.070***	0.205***	0.294***	0.033***	0.056***	-0.074***	0.036***	0.163***	0.247***	0.418***	0.038***	1.000
Observations	11128665											

Standard errors in parentheses

* $p < 0.010$, ** $p < 0.005$, *** $p < 0.001$

Table S3.2: Bivariate correlations of variables with *novelty occurrence*.

	(1)	(2)	(3)	(4)	(5)	(6)	(7)	(8)	(9)	(10)	(11)	(12)	(13)
(1) $\mathcal{N}ovelty\mathcal{B}readth$	1.000												
(2) $\mathcal{N}ovelty\mathcal{D}istance$	0.583***	1.000											
(3) $Ln(T_{size})$	-0.061***	-0.049***	1.000										
(4) $Ln(Field_{size})$	-0.021***	-0.084***	0.138***	1.000									
(5) $Ln(N_{refs})$	0.304***	-0.000	-0.165***	0.029***	1.000								
(6) $Ln(Pub_{fields})$	0.024***	-0.001	0.016***	-0.081***	0.028***	1.000							
(7) $International$	0.000	0.003	0.231***	-0.004*	0.031***	-0.004	1.000						
(8) $Ln(N_{orgs})$	-0.017***	-0.010***	0.468***	0.031***	-0.025***	0.004**	0.557***	1.000					
(9) $Ln(JIF)$	0.075***	-0.049***	0.158***	0.101***	0.275***	0.026***	0.065***	0.063***	1.000				
(10) UI	-0.004*	-0.032***	0.280***	0.066***	-0.024***	0.014***	0.195***	0.524***	0.049***	1.000			
(11) $Industry$	0.007***	-0.027***	-0.089***	0.065***	0.005**	-0.008***	-0.121***	-0.187***	-0.025***	-0.152***	1.000		
(12) $Appliedness_f$	0.017***	-0.085***	0.079***	0.221***	0.066***	-0.028***	-0.011***	-0.042***	0.197***	-0.011***	-0.019***	1.000	
(13) $IndPubIntensity_f$	0.043***	-0.100***	0.104***	0.280***	0.036***	0.130***	-0.056***	0.043***	0.011***	0.228***	0.290***	-0.033***	1.000
Observations	446,637												

Standard errors in parentheses

* $p < 0.010$, ** $p < 0.005$, *** $p < 0.001$

Table S3.3: Bivariate correlations of variables with *novelty breadth* and *novelty distance*.

S4 Probit first stage

	DV: Author affiliation classification available for all authors of a publication	
	(1)	(2)
$Ln(Field_{size})$	0.026	-0.007
	(0.020)	(0.011)
$Ln(N_{refs})$	0.088***	0.052***
	(0.007)	(0.010)
$Ln(Pub_{fields})$	0.001	-0.001
	(0.005)	(0.004)
$Appliedness_f$	0.021	0.044**
	(0.018)	(0.018)
$Ln(JIF)$	0.010	
	(0.017)	
Constant	0.438***	0.302***
	(0.025)	(0.022)
Year FE		
Observations	16,068,501	683,852
Groups	1646	1447
AIC	19703220	838996
BIC	19703512	839213

Standard errors in parentheses
* $p < 0.10$, ** $p < 0.05$, *** $p < 0.01$
All independent variables except dummy variables are standardized about the mean.
IMR: Inverse-Mill's Ratio. Year FE: Year Fixed-effects.

Table S4.1: Probit First Stage estimates.

S5 UI and sole-industry publishing contribution to a field

Industry publishing contribution to a field can arise from the concentration of two categories of publications—those authored exclusively by industry-affiliated authors and those co-authored by individuals from both university and industry (UI-authored publications). To incorporate their differential association with novelty, we conduct additional analysis by stratifying industry publishing contribution into two parts:

UI publishing contribution ($UI\ PubContribution_f$): Percentage of publications within a field with some authors affiliated with university and some with industry;

Sole-industry publishing contribution ($Sole - Ind\ PubContribution_f$): Percentage of publications within a field with all authors affiliated with industry.

Our main results of a positive association of industry publishing contribution to a field with *novelty occurrence* and *novelty distance*, and negative association with *novelty distance* remain largely consistent with these specifications (Table E1). UI publishing contribution ($UI\ PubContribution_f$) is positively associated with *novelty occurrence* and negatively associated with *novelty distance*, although the association with *novelty breadth* is not statistically significant. Similarly, sole-industry publishing contribution ($Sole - Ind\ PubContribution_f$) is positively associated with both *novelty occurrence* and *novelty breadth*, while its association with *novelty distance* is not statistically significant. These variations in statistical significance in some of the robustness tests may stem from the field-level nature of our analysis, where limited within-field variation could affect the precision of some estimates.

	(1) $Logit(\mathcal{N}ovelty\mathcal{O}ccurrence)$	(2) $Ln(\mathcal{N}ovelty\mathcal{B}readth)$	(3) $Ln(\mathcal{N}ovelty\mathcal{D}istance)$
$Ln(T_{size})$	0.032***	-0.009***	-0.006***
	(0.012)	(0.003)	(0.001)
$Ln(Field_{size})$	-0.676***	-0.152***	-0.022***
	(0.107)	(0.015)	(0.005)
$Ln(N_{refs})$	-0.052	1.201***	0.053*
	(0.274)	(0.097)	(0.029)
$Ln(Pub_{fields})$	-0.010	-0.016***	0.001
	(0.012)	(0.004)	(0.001)
$International$	-0.073***	-0.006	-0.001
	(0.010)	(0.004)	(0.001)
$Ln(N_{orgs})$	-0.002	-0.006***	0.001
	(0.010)	(0.002)	(0.001)
$Ln(JIF)$	-0.090**	-0.011**	-0.012***
	(0.039)	(0.005)	(0.002)
UI	0.038*	0.005	-0.003
	(0.020)	(0.006)	(0.002)
$Industry$	-0.109***	-0.013	-0.006***
	(0.021)	(0.009)	(0.002)
$Appliedness_f$	-0.321***	0.720***	0.032
	(0.081)	(0.082)	(0.026)
$UI\ PubContribution_f$	0.281***	0.003	-0.036***
	(0.040)	(0.008)	(0.004)
$Sole - Ind\ PubContribution_f$	0.133**	0.045***	0.008
	(0.059)	(0.008)	(0.005)
IMR	-40.792***	31.171***	1.826*
	(6.340)	(3.552)	(1.091)
Constant	17.417***	-16.778***	-1.158**
	(3.413)	(1.899)	(0.583)
Year FE	Yes	Yes	Yes
Observations	11,128,665	446,785	446,785
Groups	1639	1384	1384
AIC	3039420	1049323	120045
BIC	3039818	1049477	120199

Standard errors clustered at field level in parentheses.

* $p < 0.10$, ** $p < 0.05$, *** $p < 0.01$

All independent variables except dummy variables are standardized about the mean.

IMR: Inverse-Mill's Ratio. Year FE: Year Fixed-effects.

Table S5.1: Regression estimates examining the relationship between industry publishing contribution emanating from UI and solely-industry publications ($UI\ PubContribution_f$ and $Sole - Ind\ PubContribution_f$) and various dimensions of novelty.

S6 Estimates using 'minimum' of industry publishing contribution among a publication's fields

As publications may be associated with multiple fields, and we assign each publication a single field by selecting the field with the maximum industry publishing contribution in the main results. As a robustness check, we alternatively use the field with the minimum contribution, which provides a conservative lower bound on the estimates.

Consequently, the measure of industry publishing contribution for a publication in year y takes the following form:

$$Ind\ PubContribution_f = \min_s(\%\text{ publications with at least one industry-affiliated author in field } s \text{ in year } y)$$

where, s is the set of fields the publication belongs to, and field f assigned to a publication is given by,

$$f = \arg\min_s(\%\text{ publications with at least one industry-affiliated author in field } s \text{ in year } y)$$

Our findings remain consistent with the 'minimum' value of industry publishing contribution among the fields of publication.

	(1)	(2)	(3)	(4)	(5)	(6)	(7)	(8)	(9)
	$Logit(\mathcal{N}ovelty\mathcal{O}ccurrence)$			$Ln(\mathcal{N}ovelty\mathcal{B}readth)$			$Ln(\mathcal{N}ovelty\mathcal{D}istance)$		
$Ln(T_{size})$	0.091***	0.066***	0.059***	-0.008***	-0.009***	-0.009***	-0.010***	-0.009***	-0.008***
	(0.015)	(0.014)	(0.013)	(0.002)	(0.002)	(0.002)	(0.001)	(0.001)	(0.001)
$Ln(Field_{size})$	-0.654**	-1.023***	-0.945***	0.400***	0.391***	0.391***	0.001	0.010	0.011
	(0.271)	(0.191)	(0.184)	(0.026)	(0.027)	(0.027)	(0.014)	(0.013)	(0.012)
$Ln(N_{refs})$	0.890**	0.406	0.507*	1.753***	1.742***	1.738***	0.052	0.063*	0.067*
	(0.397)	(0.280)	(0.273)	(0.085)	(0.088)	(0.088)	(0.044)	(0.037)	(0.036)
$Ln(Pub_{fields})$	-0.003	-0.022	-0.018	0.032***	0.033***	0.033***	-0.002	-0.003**	-0.003**
	(0.027)	(0.021)	(0.020)	(0.003)	(0.003)	(0.003)	(0.001)	(0.001)	(0.001)
$International$	-0.142***	-0.104***	-0.106***	-0.011***	-0.008**	-0.008**	0.003***	0.001	0.000
	(0.015)	(0.013)	(0.012)	(0.004)	(0.004)	(0.004)	(0.001)	(0.001)	(0.001)
$Ln(N_{orgs})$	-0.023	-0.009	0.005	-0.007***	-0.006***	-0.005**	0.002***	0.001	0.000
	(0.014)	(0.012)	(0.012)	(0.002)	(0.002)	(0.002)	(0.001)	(0.001)	(0.001)
$Ln(JIF)$	-0.000	-0.059	-0.052	-0.017***	-0.015***	-0.015***	-0.013***	-0.015***	-0.015***
	(0.059)	(0.043)	(0.043)	(0.005)	(0.005)	(0.005)	(0.002)	(0.002)	(0.002)
UI	0.359***	0.153***	0.247***	0.031***	0.016***	0.023***	-0.020***	-0.004**	-0.009***
	(0.042)	(0.025)	(0.027)	(0.006)	(0.005)	(0.005)	(0.003)	(0.002)	(0.002)
$Industry$	0.272***	-0.025	0.283***	0.026***	0.005	0.026***	-0.019***	0.002	-0.020***
	(0.043)	(0.020)	(0.030)	(0.007)	(0.007)	(0.007)	(0.006)	(0.002)	(0.004)
$Appliedness_f$	0.349***	0.443***	0.399***	0.355***	0.352***	0.350***	-0.005	-0.003	-0.001
	(0.100)	(0.075)	(0.072)	(0.025)	(0.026)	(0.026)	(0.015)	(0.013)	(0.012)
$Ind\ PubContribution_f$		0.268***	0.371***		0.024***	0.032***		-0.024***	-0.031***
		(0.037)	(0.040)		(0.007)	(0.008)		(0.005)	(0.005)
$UI \times Ind\ PubContribution_f$			-0.276***			-0.021***			0.017***
			(0.027)			(0.006)			(0.003)
$Industry \times Ind\ PubContribution_f$			-0.388***			-0.030***			0.030***
			(0.041)			(0.009)			(0.005)
IMR	-18.921**	-29.830***	-27.352***	50.123***	49.786***	49.671***	1.810	2.151	2.264*
	(9.002)	(6.260)	(6.091)	(3.053)	(3.128)	(3.154)	(1.604)	(1.342)	(1.293)
Constant	5.038	10.396***	9.221***	-26.933***	-26.751***	-26.688***	-1.158	-1.341*	-1.403**
	(4.363)	(3.041)	(2.960)	(1.634)	(1.674)	(1.688)	(0.857)	(0.718)	(0.691)
Year FE	Yes	Yes	Yes	Yes	Yes	Yes	Yes	Yes	Yes
Observations	11,128,665	11,128,665	11,128,665	446,785	446,785	446,785	446,785	446,785	446,785
Groups	1663	1663	1663	1470	1470	1470	1470	1470	1470
AIC	3076309	3059362	3052815	1049618	1049376	1049313	124847	122857	122402
BIC	3076679	3059746	3053227	1049750	1049519	1049478	124979	123000	122568

Standard errors in parentheses

* $p < 0.10$, ** $p < 0.05$, *** $p < 0.01$

All independent variables except dummy variables are standardized about the mean.

IMR: Inverse-Mill's Ratio. Year FE: Year Fixed-effects.

Table S6.1: Estimates using 'minimum' of industry publishing intensity among a publication's fields.

S7 Estimates with a four-year lag for the field variables

Since our interest lies in a field-level driver of novelty, and any field-level effects require time to materialize due to delays inherent in the publication process, we lag all field-level variables by two years in the main analysis. As a robustness check, we re-estimate the models using four-year–lagged field-level variables, in Table S7.1. The results remain qualitatively consistent across these specifications.

	(1)	(2)	(3)	(4)	(5)	(6)	(7)	(8)	(9)
	$Logit(\mathcal{N}ovelty\mathcal{O}ccurrence)$			$Ln(\mathcal{N}ovelty\mathcal{B}readth)$			$Ln(\mathcal{N}ovelty\mathcal{D}istance)$		
$Ln(T_{size})$	0.096***	0.047***	0.042***	-0.006**	-0.010***	-0.010***	-0.010***	-0.008***	-0.008***
	(0.015)	(0.011)	(0.011)	(0.003)	(0.002)	(0.002)	(0.001)	(0.001)	(0.001)
$Ln(Field_{size})$	-0.524***	-0.710***	-0.713***	-0.098***	-0.109***	-0.109***	-0.027***	-0.021***	-0.021***
	(0.192)	(0.132)	(0.128)	(0.014)	(0.011)	(0.011)	(0.005)	(0.004)	(0.004)
$Ln(N_{refs})$	0.456	0.156	0.114	1.183***	1.185***	1.185***	0.084*	0.082**	0.082**
	(0.396)	(0.297)	(0.292)	(0.101)	(0.093)	(0.093)	(0.046)	(0.040)	(0.038)
$Ln(Pub_{fields})$	0.031	-0.012	-0.013	-0.005	-0.011***	-0.011***	-0.003*	-0.000	0.000
	(0.019)	(0.015)	(0.014)	(0.004)	(0.003)	(0.003)	(0.002)	(0.002)	(0.002)
$International$	-0.145***	-0.078***	-0.082***	-0.012***	-0.005	-0.005	0.004***	0.000	0.000
	(0.014)	(0.010)	(0.010)	(0.004)	(0.004)	(0.004)	(0.001)	(0.001)	(0.001)
$Ln(N_{orgs})$	-0.030**	-0.004	0.010	-0.008***	-0.006***	-0.005***	0.003***	0.002*	0.001
	(0.014)	(0.010)	(0.010)	(0.002)	(0.002)	(0.002)	(0.001)	(0.001)	(0.001)
$Ln(JIF)$	0.002	-0.030	-0.044	-0.013**	-0.011**	-0.012**	-0.015***	-0.016***	-0.015***
	(0.051)	(0.039)	(0.040)	(0.005)	(0.005)	(0.005)	(0.002)	(0.002)	(0.002)
UI	0.386***	0.043**	0.189***	0.036***	0.004	0.017***	-0.022***	-0.005**	-0.012***
	(0.045)	(0.021)	(0.026)	(0.007)	(0.006)	(0.006)	(0.004)	(0.002)	(0.003)
$Industry$	0.290***	-0.157***	0.261***	0.029***	-0.012	0.024***	-0.022***	-0.000	-0.026***
	(0.059)	(0.027)	(0.041)	(0.010)	(0.009)	(0.009)	(0.005)	(0.003)	(0.005)
$Appliedness_f$	-0.197*	-0.242***	-0.265***	0.692***	0.702***	0.702***	0.058	0.052	0.052
	(0.101)	(0.080)	(0.080)	(0.086)	(0.079)	(0.079)	(0.043)	(0.037)	(0.035)
$Ind\ PubContribution_f$		0.391***	0.470***		0.047***	0.054***		-0.026***	-0.031***
		(0.045)	(0.044)		(0.008)	(0.008)		(0.005)	(0.005)
$UI{\times}Ind\ PubContribution_f$			-0.278***			-0.025***			0.016***
			(0.020)			(0.005)			(0.004)
$Industry{\times}Ind\ PubContribution_f$			-0.444***			-0.041***			0.029***
			(0.032)			(0.009)			(0.005)
IMR	-29.803***	-36.439***	-37.233***	30.259***	30.482***	30.522***	3.106*	2.984**	2.955**
	(9.315)	(6.914)	(6.784)	(3.689)	(3.385)	(3.370)	(1.720)	(1.486)	(1.419)
Constant	10.043**	13.292***	13.695***	-16.304***	-16.424***	-16.445***	-1.849**	-1.784**	-1.769**
	(4.432)	(3.297)	(3.239)	(1.975)	(1.812)	(1.804)	(0.919)	(0.794)	(0.759)
Year FE	Yes	Yes	Yes	Yes	Yes	Yes	Yes	Yes	Yes
Observations	11,128,343	11,128,343	11,128,343	446,772	446,772	446,772	446,772	446,772	446,772
Groups	1583	1583	1583	1346	1346	1346	1346	1346	1346
AIC	3082582	3043291	3036633	1050175	1049183	1049090	125071	122716	122365
BIC	3082952	3043676	3037046	1050307	1049326	1049255	125203	122859	122530

Standard errors clustered at field level in parentheses.

* $p < 0.10$, ** $p < 0.05$, *** $p < 0.01$

All independent variables except dummy variables are standardized about the mean.

IMR: Inverse-Mill's Ratio. Year FE: Year Fixed-effects.

Table S7.1: Estimates with a four-year lag for the field variables.

S8 Alternative field classification

We assign publications to scientific fields using OpenAlex's hierarchical concepts taxonomy, which provides an associated confidence score for each concept assignment. In the main analysis, we use a confidence threshold corresponding to the median confidence score. Because field classifications may vary with the choice of confidence threshold, we conduct robustness checks using a more stringent cutoff at the 75th percentile of confidence scores across all publications when assigning Level 0 and Level 1 concepts. Under this specification, the cutoff confidence scores are 0.53 for Level 0 concepts and 0.41 for Level 1 concepts. The results from these alternative field assignments are reported in Table S8.1 and remain qualitatively consistent with the main results.

	(1)	(2)	(3)	(4)	(5)	(6)	(7)	(8)	(9)
	$Logit(\mathcal{N}ovelty\mathcal{O}ccurrence)$			$Ln(\mathcal{N}ovelty\mathcal{B}readth)$			$Ln(\mathcal{N}ovelty\mathcal{D}istance)$		
$Ln(T_{size})$	0.088***	0.055***	0.050***	-0.008***	-0.009***	-0.010***	-0.007***	-0.006***	-0.006***
	(0.018)	(0.013)	(0.013)	(0.003)	(0.003)	(0.003)	(0.002)	(0.001)	(0.001)
$Ln(Field_{size})$	-0.327**	-0.485***	-0.487***	-0.040***	-0.047***	-0.047***	-0.014***	-0.010**	-0.010**
	(0.165)	(0.125)	(0.119)	(0.011)	(0.009)	(0.009)	(0.005)	(0.005)	(0.005)
$Ln(N_{refs})$	0.911***	0.658**	0.623**	1.029***	1.021***	1.021***	0.057*	0.061**	0.062**
	(0.325)	(0.292)	(0.276)	(0.087)	(0.089)	(0.089)	(0.033)	(0.029)	(0.029)
$Ln(Pub_{fields})$	0.115**	0.138***	0.145***	-0.172***	-0.174***	-0.174***	-0.015*	-0.014*	-0.014*
	(0.053)	(0.049)	(0.047)	(0.022)	(0.022)	(0.022)	(0.009)	(0.008)	(0.007)
$International$	-0.141***	-0.083***	-0.087***	-0.011*	-0.006	-0.006	0.005***	0.002	0.002
	(0.018)	(0.014)	(0.014)	(0.006)	(0.006)	(0.006)	(0.002)	(0.002)	(0.002)
$Ln(N_{orgs})$	-0.026	-0.005	0.009	-0.008***	-0.006***	-0.005**	0.001	-0.000	-0.001
	(0.016)	(0.012)	(0.013)	(0.002)	(0.002)	(0.002)	(0.001)	(0.001)	(0.001)
$Ln(JIF)$	0.193***	0.249***	0.246***	-0.021***	-0.017***	-0.017***	-0.012***	-0.014***	-0.014***
	(0.067)	(0.063)	(0.061)	(0.006)	(0.006)	(0.006)	(0.002)	(0.002)	(0.002)
UI	0.401***	0.089***	0.183***	0.041***	0.014	0.019**	-0.020***	-0.004	-0.008**
	(0.051)	(0.027)	(0.028)	(0.009)	(0.009)	(0.008)	(0.005)	(0.002)	(0.003)
$Industry$	0.319***	-0.103***	0.218***	0.031**	-0.005	0.029***	-0.022***	0.000	-0.018***
	(0.060)	(0.028)	(0.043)	(0.013)	(0.013)	(0.011)	(0.006)	(0.003)	(0.006)
$Appliedness_f$	0.065	0.028	0.007	0.513***	0.511***	0.510***	0.022	0.023	0.024
	(0.058)	(0.059)	(0.059)	(0.063)	(0.066)	(0.066)	(0.028)	(0.024)	(0.023)
$Ind\ PubContribution_f$		0.348***	0.433***		0.036***	0.044***		-0.022***	-0.027***
		(0.048)	(0.047)		(0.009)	(0.009)		(0.005)	(0.005)
$UI \times Ind\ PubContribution_f$			-0.256***			-0.019**			0.013***
			(0.027)			(0.008)			(0.005)
$Industry \times Ind\ PubContribution_f$			-0.381***			-0.041***			0.023***
			(0.039)			(0.013)			(0.007)
IMR	-24.358**	-32.647***	-33.636***	29.915***	29.667***	29.680***	2.443*	2.595**	2.588**
	(11.006)	(9.764)	(9.235)	(3.724)	(3.823)	(3.816)	(1.440)	(1.267)	(1.239)
Constant	6.908	10.754**	11.235***	-15.878***	-15.746***	-15.751***	-1.481*	-1.562**	-1.559**
	(4.981)	(4.430)	(4.191)	(1.965)	(2.017)	(2.013)	(0.759)	(0.668)	(0.653)
Year FE	Yes	Yes	Yes	Yes	Yes	Yes	Yes	Yes	Yes
Observations	6,570,778	6,570,778	6,570,778	239,427	239,427	239,427	239,427	239,427	239,427
Groups	617	617	617	551	551	551	551	551	551
AIC	1705965	1690300	1687355	552186	551859	551811	75083	74170	74045
BIC	1706321	1690670	1687753	552311	551994	551966	75208	74305	74201

Standard errors in parentheses

* $p < 0.10$, ** $p < 0.05$, *** $p < 0.01$

All independent variables except dummy variables are standardized about the mean.

IMR: Inverse-Mill's Ratio. Year FE: Year Fixed-effects.

Table S8.1: Estimates using alternative field classification.

S9 Alternative author classification

We also use an alternate broader classification for university, where we include authors affiliated with the categories ‘education’, ‘facility’ and ‘nonprofit’ as university authors (see section S2) and authors from with the categories of ‘company’, ‘healthcare’ and ‘archive’ as industry-affiliated authors.

	(1)	(2)	(3)	(4)	(5)	(6)	(7)	(8)	(9)
	$Logit(\mathcal{N}ovelty\mathcal{O}ccurrence)$			$Ln(\mathcal{N}ovelty\mathcal{B}readth)$			$Ln(\mathcal{N}ovelty\mathcal{D}istance)$		
$Ln(T_{size})$	0.086***	0.039***	0.034***	-0.007**	-0.010***	-0.010***	-0.010***	-0.008***	-0.008***
	(0.015)	(0.011)	(0.011)	(0.003)	(0.003)	(0.003)	(0.001)	(0.001)	(0.001)
$Ln(Field_{size})$	-0.492***	-0.620***	-0.617***	-0.100***	-0.110***	-0.109***	-0.026***	-0.020***	-0.020***
	(0.109)	(0.080)	(0.080)	(0.014)	(0.011)	(0.011)	(0.004)	(0.004)	(0.004)
$Ln(N_{refs})$	0.214	0.064	0.036	1.057***	1.048***	1.048***	0.064**	0.071***	0.071***
	(0.216)	(0.203)	(0.209)	(0.079)	(0.076)	(0.076)	(0.027)	(0.024)	(0.023)
$Ln(Pub_{fields})$	0.029*	-0.010	-0.011	-0.008*	-0.013***	-0.013***	-0.003*	0.000	0.000
	(0.015)	(0.012)	(0.012)	(0.004)	(0.003)	(0.003)	(0.002)	(0.002)	(0.002)
$International$	-0.144***	-0.078***	-0.082***	-0.012***	-0.006	-0.006	0.003*	-0.001	-0.001
	(0.013)	(0.010)	(0.010)	(0.004)	(0.004)	(0.004)	(0.001)	(0.001)	(0.001)
$Ln(N_{orgs})$	-0.020	0.001	0.014	-0.008***	-0.006***	-0.005***	0.003***	0.002*	0.001
	(0.014)	(0.010)	(0.010)	(0.002)	(0.002)	(0.002)	(0.001)	(0.001)	(0.001)
$Ln(JIF)$	-0.521***	-0.581***	-0.600***	-0.013**	-0.012**	-0.013**	-0.014***	-0.015***	-0.015***
	(0.105)	(0.095)	(0.098)	(0.005)	(0.005)	(0.005)	(0.002)	(0.002)	(0.002)
UI	0.360***	0.030	0.187***	0.034***	0.005	0.020***	-0.021***	-0.002	-0.010***
	(0.044)	(0.020)	(0.025)	(0.007)	(0.007)	(0.006)	(0.004)	(0.002)	(0.003)
$Industry$	0.275***	-0.156***	0.264***	0.027***	-0.008	0.025***	-0.022***	0.002	-0.024***
	(0.059)	(0.029)	(0.038)	(0.010)	(0.009)	(0.009)	(0.005)	(0.003)	(0.005)
$Appliedness_f$	-0.389***	-0.420***	-0.441***	0.625***	0.622***	0.622***	0.042	0.043*	0.044*
	(0.082)	(0.081)	(0.083)	(0.071)	(0.068)	(0.068)	(0.027)	(0.024)	(0.023)
$Ind\ PubContribution_f$		0.382***	0.457***		0.041***	0.048***		-0.027***	-0.032***
		(0.044)	(0.043)		(0.008)	(0.008)		(0.004)	(0.004)
$UI \times Ind\ PubContribution_f$			-0.273***			-0.025***			0.015***
			(0.020)			(0.006)			(0.004)
$Industry \times Ind\ PubContribution_f$			-0.435***			-0.037***			0.028***
			(0.031)			(0.009)			(0.006)
IMR	-34.819***	-37.759***	-38.241***	20.259***	20.076***	20.091***	1.869**	1.991***	1.980***
	(4.978)	(4.583)	(4.736)	(2.270)	(2.168)	(2.170)	(0.792)	(0.719)	(0.690)
Constant	11.055***	12.415***	12.646***	-9.911***	-9.823***	-9.830***	-1.092***	-1.150***	-1.146***
	(2.179)	(2.011)	(2.079)	(1.098)	(1.049)	(1.050)	(0.383)	(0.348)	(0.334)
Year FE	Yes	Yes	Yes	Yes	Yes	Yes	Yes	Yes	Yes
Observations	11,568,145	11,568,145	11,568,145	473,394	473,394	473,394	473,394	473,394	473,394
Groups	1636	1636	1636	1387	1387	1387	1387	1387	1387
AIC	3253139	3213175	3206700	1113491	1112676	1112592	136403	133562	133241
BIC	3253510	3213560	3207113	1113623	1112820	1112758	136536	133706	133407

Standard errors in parentheses

* $p < 0.10$, ** $p < 0.05$, *** $p < 0.01$

All independent variables except dummy variables are standardized about the mean.

IMR: Inverse-Mill’s Ratio. Year FE: Year Fixed-effects.

Table S9.1: Estimates using alternative author classification.

S10 Relaxing the Journal Impact-Factor restriction for Novelty calculation

In the main analysis, we restrict attention to novel recombinations involving journals with impact factors above the 75th percentile, with the aim of ensuring substantive and credible instances of knowledge recombination. As a robustness check, we relax this restriction and re-estimate the models using the full set of novel journal pairs, irrespective of journal impact factor in Table S10.1. The results are qualitatively similar across specifications, indicating that the findings are not sensitive to the chosen journal impact-factor threshold.

	(1)	(2)	(3)	(4)	(5)	(6)	(7)	(8)	(9)
	$Logit(\mathcal{N}ovelty\mathcal{O}ccurrence)$			$Ln(\mathcal{N}ovelty\mathcal{B}readth)$			$Ln(\mathcal{N}ovelty\mathcal{D}istance)$		
$Ln(T_{size})$	-0.021*	-0.053***	-0.056***	-0.009**	-0.013***	-0.013***	-0.004*	-0.002	-0.002
	(0.012)	(0.011)	(0.011)	(0.004)	(0.004)	(0.004)	(0.002)	(0.002)	(0.002)
$Ln(Field_{size})$	-0.345**	-0.457***	-0.465***	0.005	-0.004	-0.004	-0.035***	-0.030***	-0.030***
	(0.137)	(0.116)	(0.116)	(0.013)	(0.012)	(0.012)	(0.005)	(0.006)	(0.006)
$Ln(N_{refs})$	0.664*	0.454	0.405	2.163***	2.145***	2.144***	-0.002	0.009	0.009
	(0.376)	(0.340)	(0.343)	(0.187)	(0.185)	(0.186)	(0.057)	(0.055)	(0.054)
$Ln(Pub_{fields})$	0.011	-0.012	-0.013	0.008*	0.004	0.004	0.003	0.005**	0.005**
	(0.013)	(0.011)	(0.011)	(0.004)	(0.003)	(0.003)	(0.002)	(0.002)	(0.002)
$International$	-0.089***	-0.050***	-0.053***	-0.005	0.001	0.001	-0.002	-0.005***	-0.005***
	(0.013)	(0.010)	(0.009)	(0.004)	(0.004)	(0.004)	(0.002)	(0.002)	(0.002)
$Ln(N_{orgs})$	0.010	0.024**	0.031***	-0.005**	-0.003	-0.003	0.004***	0.003***	0.003***
	(0.012)	(0.010)	(0.010)	(0.002)	(0.002)	(0.002)	(0.001)	(0.001)	(0.001)
$Ln(JIF)$	-0.351***	-0.378***	-0.387***	-0.057***	-0.057***	-0.058***	-0.027***	-0.026***	-0.026***
	(0.048)	(0.045)	(0.046)	(0.006)	(0.006)	(0.006)	(0.004)	(0.004)	(0.004)
UI	0.197***	-0.001	0.068***	0.015	-0.011**	-0.004	-0.022***	-0.006**	-0.004
	(0.036)	(0.016)	(0.020)	(0.009)	(0.005)	(0.005)	(0.006)	(0.002)	(0.003)
$Industry$	0.118***	-0.158***	0.036	0.013	-0.023***	-0.006	-0.044***	-0.022***	-0.030***
	(0.039)	(0.018)	(0.025)	(0.012)	(0.007)	(0.008)	(0.009)	(0.003)	(0.005)
$Appliedness_f$	-0.152*	-0.196**	-0.216**	0.778***	0.772***	0.771***	-0.030	-0.027	-0.027
	(0.090)	(0.086)	(0.088)	(0.089)	(0.088)	(0.089)	(0.029)	(0.027)	(0.027)
$Ind\ PubContribution_f$		0.217***	0.259***		0.032***	0.036***		-0.019***	-0.020***
		(0.033)	(0.036)		(0.010)	(0.010)		(0.006)	(0.006)
$UI \times Ind\ PubContribution_f$			-0.158***			-0.015**			-0.002
			(0.022)			(0.007)			(0.005)
$Industry \times Ind\ PubContribution_f$			-0.216***			-0.019*			0.008
			(0.030)			(0.011)			(0.007)
IMR	-16.308*	-20.993***	-22.019***	37.713***	37.372***	37.343***	-0.625	-0.418	-0.410
	(8.357)	(7.537)	(7.605)	(3.962)	(3.938)	(3.949)	(1.228)	(1.175)	(1.173)
Constant	5.593	7.896**	8.402**	-20.532***	-20.340***	-20.323***	-0.012	-0.129	-0.133
	(3.990)	(3.603)	(3.637)	(2.154)	(2.141)	(2.147)	(0.670)	(0.640)	(0.639)
Year FE	Yes	Yes	Yes	Yes	Yes	Yes	Yes	Yes	Yes
Observations	11,128,665	11,128,665	11,128,665	1,472,632	1,472,632	1,472,632	1,472,632	1,472,632	1,472,632
Groups	1639	1639	1639	1565	1565	1565	1565	1565	1565
AIC	7587953	7553331	7548295	3873397	3872231	3872173	635989	632099	632036
BIC	7588323	7553715	7548708	3873544	3872390	3872356	636136	632258	632219

Standard errors clustered at field level in parentheses.

* $p < 0.10$, ** $p < 0.05$, *** $p < 0.01$

All independent variables except dummy variables are standardized about the mean.

IMR: Inverse-Mill's Ratio. Year FE: Year Fixed-effects.

Table S10.1: Regression estimates without Journal Impact Factor restriction for novel journal pairs.

S11 Alternative specification for industry publishing contribution

In our main analysis, we measure industry publishing contribution to a field as the percentage of industry-authored publications within that field. To assess the robustness of our findings to alternative operationalizations, we additionally conduct the analysis using two alternative measures of industry publishing contribution to a field.

S11.1 Percentage of industry among all institutions within a field

As a first alternative, we measure industry publishing contribution as the share of industry firms among all publishing institutions within a field. Specifically, we calculate the proportion of distinct institutional affiliations classified as industry relative to the total number of distinct institutions that publish in the field. The estimates remain consistent with the main results.

	(1)	(2)	(3)	(4)	(5)	(6)
	$Logit(\mathcal{N}ovelty\mathcal{O}ccurrence)$		$Ln(\mathcal{N}ovelty\mathcal{B}readth)$		$Ln(\mathcal{N}ovelty\mathcal{D}istance)$	
$Ln(T_{size})$	0.042***	0.038***	-0.010***	-0.010***	-0.009***	-0.008***
	(0.011)	(0.011)	(0.002)	(0.002)	(0.001)	(0.001)
$Ln(Field_{size})$	-0.715***	-0.726***	-0.156***	-0.156***	-0.016***	-0.016***
	(0.147)	(0.144)	(0.017)	(0.016)	(0.005)	(0.005)
$Ln(N_{refs})$	0.172	0.106	1.197***	1.199***	0.062**	0.060**
	(0.328)	(0.326)	(0.097)	(0.097)	(0.030)	(0.029)
$Ln(Pub_{fields})$	0.001	0.000	-0.015***	-0.015***	-0.000	-0.000
	(0.016)	(0.016)	(0.004)	(0.004)	(0.002)	(0.002)
International	-0.065***	-0.068***	-0.006	-0.006	-0.000	-0.000
	(0.010)	(0.010)	(0.004)	(0.004)	(0.001)	(0.001)
$Ln(N_{orgs})$	-0.001	0.011	-0.006***	-0.006***	0.002*	0.001
	(0.009)	(0.009)	(0.002)	(0.002)	(0.001)	(0.001)
$Ln(JIF)$	-0.042	-0.059	-0.013**	-0.013**	-0.015***	-0.015***
	(0.043)	(0.044)	(0.005)	(0.005)	(0.002)	(0.002)
UI	0.025	0.170***	0.008	0.020***	-0.004**	-0.012***
	(0.018)	(0.026)	(0.007)	(0.007)	(0.002)	(0.003)
Industry	-0.177***	0.259***	-0.006	0.031***	0.000	-0.027***
	(0.030)	(0.044)	(0.009)	(0.011)	(0.003)	(0.005)
$Appliedness_f$	-0.238***	-0.269***	0.712***	0.712***	0.033	0.033
	(0.077)	(0.077)	(0.083)	(0.083)	(0.028)	(0.028)
$\%\ Ind\ Orgs_f$	0.467***	0.536***	0.045***	0.051***	-0.030***	-0.035***
	(0.045)	(0.043)	(0.007)	(0.007)	(0.005)	(0.005)
$UI{\times}\%\ Ind\ Orgs_f$		-0.269***		-0.022***		0.015***
		(0.020)		(0.007)		(0.004)
$Industry{\times}\%\ Ind\ Orgs_f$		-0.464***		-0.042***		0.031***
		(0.036)		(0.011)		(0.005)
IMR	-35.657***	-37.072***	31.025***	31.104***	2.183*	2.126*
	(7.618)	(7.563)	(3.578)	(3.580)	(1.135)	(1.097)
Constant	12.984***	13.667***	-16.704***	-16.745***	-1.354**	-1.324**
	(3.630)	(3.608)	(1.913)	(1.914)	(0.607)	(0.587)
Year FE	Yes	Yes	Yes	Yes	Yes	Yes
Observations	11,120,514	11,120,514	446,501	446,501	446,501	446,501
Groups	1635	1635	1380	1380	1380	1380
AIC	3034495	3028925	1049008	1048941	122598	122304
BIC	3034879	3029338	1049151	1049106	122741	122469

Standard errors in parentheses
* $p < 0.10$, ** $p < 0.05$, *** $p < 0.01$
All independent variables except dummy variables are standardized about the mean.
IMR: Inverse-Mill's Ratio. Year FE: Year Fixed-effects.

Table S11.1: Regression estimates with industry publishing contribution measured as the share of industry institutions within a field.

S11.2 Ratio of industry to university publications within a field

As a second alternative, we operationalize industry publishing contribution as the ratio of industry-authored publications to university-authored publications within a field. This measure reflects the relative intensity of industry publishing compared to academic publishing and emphasizes the balance between industry and university knowledge production within the field. The estimates remain consistent with the main results.

	(1)	(2)	(3)	(4)	(5)	(6)
	$Logit(\mathcal{N}ovelty\mathcal{O}ccurrence)$		$Ln(\mathcal{N}ovelty\mathcal{B}readth)$		$Ln(\mathcal{N}ovelty\mathcal{D}istance)$	
$Ln(T_{size})$	0.052***	0.045***	-0.010***	-0.011***	-0.009***	-0.008***
	(0.012)	(0.012)	(0.002)	(0.003)	(0.001)	(0.001)
$Ln(Field_{size})$	-0.639***	-0.646***	-0.152***	-0.151***	-0.021***	-0.021***
	(0.119)	(0.113)	(0.015)	(0.015)	(0.005)	(0.005)
$Ln(N_{refs})$	0.157	0.100	1.205***	1.203***	0.055*	0.056*
	(0.277)	(0.271)	(0.095)	(0.095)	(0.032)	(0.031)
$Ln(Pub_{fields})$	0.005	0.002	-0.016***	-0.017***	-0.000	-0.000
	(0.015)	(0.013)	(0.004)	(0.004)	(0.002)	(0.002)
$International$	-0.089***	-0.093***	-0.006	-0.006	0.001	0.001
	(0.011)	(0.011)	(0.004)	(0.004)	(0.001)	(0.001)
$Ln(N_{orgs})$	-0.007	0.009	-0.006***	-0.005**	0.002*	0.001
	(0.011)	(0.011)	(0.002)	(0.002)	(0.001)	(0.001)
$Ln(JIF)$	-0.032	-0.048	-0.011**	-0.012**	-0.016***	-0.015***
	(0.039)	(0.040)	(0.005)	(0.005)	(0.002)	(0.002)
UI	0.093***	0.213***	0.006	0.017***	-0.007***	-0.014***
	(0.025)	(0.031)	(0.006)	(0.006)	(0.002)	(0.003)
$Industry$	-0.108***	0.271***	-0.011	0.021**	-0.003	-0.026***
	(0.024)	(0.046)	(0.009)	(0.009)	(0.002)	(0.005)
$Appliedness_f$	-0.219***	-0.245***	0.721***	0.719***	0.027	0.028
	(0.074)	(0.074)	(0.081)	(0.081)	(0.030)	(0.029)
$(\#Ind\ /\ \#Uni)_f$	0.334***	0.432***	0.043***	0.053***	-0.022***	-0.028***
	(0.050)	(0.051)	(0.008)	(0.008)	(0.005)	(0.005)
$UI \times (\#Ind\ /\ \#Uni)_f$		-0.283***		-0.027***		0.017***
		(0.020)		(0.005)		(0.004)
$Industry \times (\#Ind\ /\ \#Uni)_f$		-0.432***		-0.040***		0.029***
		(0.032)		(0.009)		(0.005)
IMR	-36.388***	-37.508***	31.317***	31.266***	1.950	1.983*
	(6.394)	(6.245)	(3.486)	(3.482)	(1.189)	(1.146)
Constant	13.253***	13.820***	-16.857***	-16.829***	-1.230*	-1.249**
	(3.051)	(2.984)	(1.864)	(1.862)	(0.635)	(0.613)
Year FE	Yes	Yes	Yes	Yes	Yes	Yes
Observations	11,128,665	11,128,665	446,785	446,785	446,785	446,785
Groups	1639	1639	1384	1384	1384	1384
AIC	3053616	3045692	1049395	1049283	123357	122913
BIC	3054000	3046105	1049538	1049448	123500	123078

Standard errors in parentheses

* $p < 0.10$, ** $p < 0.05$, *** $p < 0.01$

All independent variables except dummy variables are standardized about the mean.

IMR: Inverse-Mill's Ratio. Year FE: Year Fixed-effects.

Table S11.2: Regression estimates with industry publishing contribution measured as the ratio of industry to university publications within a field.

S12 Institution Rank

As an additional analysis, we explore whether the relationship between industry publishing contribution and the three novelty dimensions differs for publications affiliated with top-500 research institutions compared to other institutions (Table 3). We also undertake the analysis using the top 100 research institutions versus others (Table L1) and find that the results remain consistent.

	(1)	(2)	(3)	(4)	(5)	(6)
	$Logit(\mathcal{N}ovelty\mathcal{O}ccurrence)$		$Ln(\mathcal{N}ovelty\mathcal{B}readth)$		$Ln(\mathcal{N}ovelty\mathcal{D}istance)$	
$Ln(T_{size})$	0.044***	0.044***	-0.011***	-0.011***	-0.004***	-0.004***
	(0.011)	(0.012)	(0.002)	(0.001)	(0.001)	(0.001)
$Ln(Field_{size})$	-0.661***	-0.661***	-0.176***	-0.176***	-0.024**	-0.025**
	(0.113)	(0.113)	(0.032)	(0.012)	(0.010)	(0.010)
$Ln(N_{refs})$	0.096	0.096	1.311***	1.311***	0.070***	0.070***
	(0.273)	(0.273)	(0.103)	(0.028)	(0.022)	(0.022)
$Ln(Pub_{fields})$	-0.003	-0.003	-0.023***	-0.023***	0.000	0.000
	(0.014)	(0.014)	(0.003)	(0.001)	(0.001)	(0.001)
$International$	-0.075***	-0.075***	-0.002	-0.002	0.001	0.001
	(0.011)	(0.010)	(0.004)	(0.004)	(0.001)	(0.001)
$Ln(N_{orgs})$	0.002	0.003	-0.002	-0.002	0.000	0.000
	(0.010)	(0.010)	(0.002)	(0.002)	(0.001)	(0.001)
$Ln(JIF)$	-0.040	-0.040	-0.006	-0.006***	-0.003***	-0.003***
	(0.039)	(0.039)	(0.004)	(0.002)	(0.001)	(0.001)
UI	0.042**	0.041**	0.003	0.003	-0.004***	-0.005***
	(0.021)	(0.020)	(0.006)	(0.004)	(0.002)	(0.002)
$Industry$	-0.158***	-0.157***	-0.010	-0.010**	-0.004*	-0.004*
	(0.027)	(0.027)	(0.008)	(0.004)	(0.002)	(0.002)
$Appliedness_f$	-0.243***	-0.243***	0.839***	0.839***	0.052***	0.053***
	(0.075)	(0.075)	(0.090)	(0.024)	(0.019)	(0.019)
$Ind\ PubContribution_f$	0.384***	0.382***	0.100***	0.100***	-0.030***	-0.031***
	(0.045)	(0.046)	(0.027)	(0.013)	(0.009)	(0.009)
Top 100	-0.067***	-0.069***	-0.010***	-0.011***	0.004**	0.003**
	(0.011)	(0.013)	(0.003)	(0.003)	(0.002)	(0.001)
Top 100$\times Ind\ PubContribution_f$		0.009		0.000		0.004**
		(0.016)		(0.003)		(0.002)
IMR	-37.639***	-37.639***	35.184***	35.184***	2.109***	2.120***
	(6.287)	(6.286)	(3.771)	(1.041)	(0.803)	(0.803)
Constant	13.881***	13.882***	-18.952***	-18.952***	-1.337***	-1.342***
	(3.001)	(3.001)	(2.015)	(0.557)	(0.429)	(0.430)
Year FE	Yes	Yes	Yes	Yes	Yes	Yes
Observations	11,128,665	11,128,665	446,637	446,637	446,637	446,637
Groups	1639	1639	1236		1236	1236
AIC	3044231	3044226	1043751	1043755	110565	110549
BIC	3044629	3044639	1043894	1043920	110708	110703

Standard errors in parentheses

* $p < 0.10$, ** $p < 0.05$, *** $p < 0.01$

All independent variables except dummy variables are standardized about the mean.

IMR: Inverse-Mill's Ratio. Year FE: Year Fixed-effects.

Table S12.1: Regression estimates of the relationship between industry publishing contribution and novelty dimensions across top-500 (Top 100) and other research institutions.

References

[1] Frank van der Wouden and Hyejin Youn. "The impact of geographical distance on learning through collaboration". In: *Research Policy* 52.2 (2023), p. 104698.

[2] Charles J Gomez, Dahlia Lieberman, and Elina I Mäkinen. "Hedgehogs, foxes, and global science ecosystems: Decoding universities' research profiles across fields with nested ecological networks". In: *Research Policy* 53.7 (2024), p. 105040.

[3] Richard Florida and WM Cohen. "Engine or lnfrastructure?" In: *The University Role in Economic Development) n: Branscomb, LM, Kodama, F., Florida, R. Eds.,) ndustrializing Knowledge: University-Industry Linkages in Japan and the United States. M) T Press, London* (1999).

[4] Philippe Aghion, Mathias Dewatripont, and Jeremy C Stein. "Academic freedom, private-sector focus, and the process of innovation". In: *The RAND Journal of Economics* 39.3 (2008), pp. 617–635.

[5] Partha Dasgupta and Paul A David. "Toward a new economics of science". In: *Research Policy* 23.5 (1994), pp. 487–521.

[6] David Blumenthal et al. "University-industry research relationships in biotechnology: Implications for the university". In: *Science* 232.4756 (1986), pp. 1361–1366.

[7] Michaël Bikard, Keyvan Vakili, and Florenta Teodoridis. "When collaboration bridges institutions: The impact of university–industry collaboration on academic productivity". In: *Organization Science* 30.2 (2019), pp. 426–445.

[8] Mike Thelwall and Nabeil Maflahi. "Academic collaboration rates and citation associations vary substantially between countries and fields". In: *Journal of the Association for Information Science and Technology* 71.8 (2020), pp. 968–978.

[9] You-Na Lee, John P Walsh, and Jian Wang. "Creativity in scientific teams: Unpacking novelty and impact". In: *Research Policy* 44.3 (2015), pp. 684–697.

[10] Mario Coccia and Lili Wang. "Evolution and convergence of the patterns of international scientific collaboration". In: *Proceedings of the National Academy of Sciences* 113.8 (2016), pp. 2057–2061.

[11] Svein Kyvik and Ingvild Reymert. "Research collaboration in groups and networks: differences across academic fields". In: *Scientometrics* 113 (2017), pp. 951–967.

[12] Caroline S Wagner, Travis A Whetsell, and Satyam Mukherjee. "International research collaboration: Novelty, conventionality, and atypicality in knowledge recombination". In: *Research Policy* 48.5 (2019), pp. 1260–1270.

[13] Giovanni Abramo, Ciriaco Andrea D'Angelo, and Gianluca Murgia. "The collaboration behaviors of scientists in Italy: A field level analysis". In: *Journal of Informetrics* 7.2 (2013), pp. 442–454.

[14] Na Zhang et al. "The role of inter-and intra-organisational networks in innovation: towards requisite variety". In: *Scientometrics* (2023), pp. 1–20.

[15] Jian Wang, Reinhilde Veugelers, and Paula Stephan. "Bias against novelty in science: A cautionary tale for users of bibliometric indicators". In: *Research Policy* 46.8 (2017), pp. 1416–1436.

[16] J Heckman. "Sample selection bias as a specification error". In: *Econometrica* (1979).
